# Beyond Resource Expansion: Building Resilient Hospital Surge Capacity for Sustained Crisis Response

**DOI:** 10.3390/healthcare14203503

**Published:** 2026-10-18

**Authors:** Xiao Li, Jun Xu, Xiulai Wang, Yutao Zhang

**Affiliations:** 1 School of Management Science and Engineering, Nanjing University of Information Science and Technology, Nanjing 210044, China; 2 Jinling Hospital, Affiliated Hospital of Medical School, Nanjing University, Nanjing 210002, China

**Keywords:** hospital surge capacity, emergency response, resilience, FRAM, BN

## Abstract

**Background:** Hospital surge-capacity assessment has traditionally emphasized the expansion of beds, staff, equipment, and medical supplies, but provides limited insight into how these resources are transformed into sustained operational capabilities under uncertainty. This study develops a resilience-oriented function–capability framework to assess hospital surge capacity from a systems perspective. **Methods:** Hospital surge capacity theory and hospital resilience theory were integrated to define 5 capability dimensions: functional reconfiguration, resource configuration, operational continuity, regional coordination, and collaborative optimization. FRAM was used to model 24 operational functions and their couplings under 4 synthesized surge scenarios. An improved K-shell algorithm quantified structural importance, while Monte Carlo derived functional-performance uncertainty was represented by truncated-normal prior distributions. Bayesian network was then used for performance assessment, propagation, sensitivity, and robustness analyses. **Results:** The estimated overall surge-capacity performance was 0.5479, 0.5631, 0.5723, and 0.5867 for *S*_1_–*S*_4_, respectively. Influential functional nodes differed across scenarios, whereas operational continuity capability consistently showed the greatest sensitivity to overall performance. Cumulative 50% degradation of structurally important functions reduced overall surge capacity by 23.54%, 16.91%, 20.51%, and 19.75% in *S*_1_–*S*_4_, respectively. Robustness analyses showed that the main scenario ordering and principal influential nodes remained generally stable under alternative modeling assumptions. **Conclusions:** Hospital surge capacity depends not only on resource availability but also on the coordinated performance of interconnected operational functions. The proposed FRAM-K-shell-BN framework separates structural importance from functional-performance uncertainty and links function level variability to capability level and overall system performance, providing a structured approach for assessing hospital surge capacity under uncertain emergency conditions.

## 1. Introduction

Major disasters, including public health emergencies, earthquakes, floods, industrial accidents, and other extreme events, often lead to sudden surges in demand for emergency medical services (EMS) [1,2]. When emergency demand increases substantially and exceeds the capacity that hospitals can accommodate under existing conditions, hospitals need to expand their service capacity to meet the additional demand. Under such circumstances, hospitals must rapidly expand their operational capacity while maintaining essential healthcare services in highly uncertain and dynamic environments. Hospital surge capacity is therefore a critical component of disaster response, involving the rapid mobilization, reconfiguration, and coordination of diverse resources, organizational units, and operational activities [3,4,5]. From a systems perspective, hospitals are inherently complex sociotechnical systems, and hospital surge capacity entails continuous interactions among healthcare personnel, physical resources, technologies, information, and organizational components [6]. Consequently, hospital surge during major disasters is not simply a temporary increase in resource availability; it is a complex and dynamic process through which hospitals must maintain essential services while continuously adapting their operations to changing conditions.

Existing studies have extensively investigated hospital surge capacity from the perspectives of resource allocation, bed expansion, workforce deployment, medical supply support, and emergency preparedness. These studies have provided valuable insights into how hospitals can increase available resources and improve operational efficiency during disasters [7,8,9,10]. However, most existing approaches primarily focus on the quantity, availability, or allocation outcomes of emergency resources. Although these indicators are important for determining whether hospitals can respond to an immediate increase in demand, they provide limited insight into whether newly mobilized resources can be effectively integrated into hospital operations and sustained as emergency conditions evolve.

In disaster response, surge capacity cannot be achieved simply by adding resources to the existing healthcare system. Instead, hospitals must continuously adapt their organizational structures, coordinate operational functions, and integrate heterogeneous resources in response to evolving emergency demands in order to enhance surge capacity [11,12,13]. During emergency response, surge capacity requires not only additional physical space and personnel but also the integration of functions such as emergency command, patient triage, resource deployment, and logistical support. These functions are interdependent: changes in one function may alter the conditions, resources, timing, or outputs of other functions, producing reinforcing, compensatory, or constraining effects throughout the system [14]. Therefore, the effectiveness of surge capacity depends not only on how much additional capacity can be created, but also on whether that capacity can be functionally integrated and sustained over the course of crisis response [15].

Resilience theory provides an appropriate perspective for explaining how such surge capacity is generated [16]. Rather than emphasizing the quantity of available resources, resilience focuses on how complex systems maintain critical functions, adapt to disruptions, reorganize operational structures, and sustain system performance under changing environmental conditions [17,18]. However, hospitals need to consider their development from a long-term resilience perspective. Their core mission is to continuously provide safe, essential, and accessible healthcare services while maintaining the capacity to respond to emergencies, thereby fulfilling their fundamental responsibility to save lives and protect public health [19]. Therefore, hospitals need to align their actions with their mission and strategic priorities and, from a long-term development perspective, shift from passively utilizing available resources to proactively creating response options. This requires flexible organizational structures, resource mobilization mechanisms, and operational configurations to better address and mitigate surge capacity challenges. These missions and strategic priorities define the essential services and functions that need to be maintained, adapted, or transformed as emergency conditions evolve over time. When existing organizational arrangements and operational configurations are no longer sufficient to sustain essential healthcare services under changing emergency conditions, hospitals may need to change how resources, organizational structures, and operational functions are arranged and coordinated to achieve a more sustainable system state. This perspective is consistent with the concept of “transformative resilience” [20]. From this perspective, hospital surge capacity can be understood as a resilience-oriented systemic process in which heterogeneous system components collectively support EMS through coordinated functional activities [21,22]. This perspective further suggests that the assessment of hospital surge capacity should focus on the key functional dimensions that characterize resilient system performance, rather than relying solely on resource-based indicators [23].

Despite growing attention to hospital surge capacity and resilience, an important research gap remains in understanding how surge resources are transformed into system capabilities that enable hospitals to fulfill their core missions under prolonged and evolving disruptions. Existing studies on surge capacity rarely establish explicit relationships between operational functions and resilience-oriented system performance, while resilience assessment studies often evaluate organizational resilience without adequately representing the functional characteristics of the surge process [24,25]. Moreover, current system modeling approaches in this field primarily focus on aggregating predefined indicators into composite scores, while overlooking how system functions are coupled to support more effective performance [26]. Therefore, hospital surge systems still lack a systematic representation framework capable of linking operational functions to system-level emergency capabilities. Such a framework should represent how operational functions contribute to different dimensions of hospital surge capacity.

To address this gap, a systems perspective is needed to bridge the relationship between operational functions and capability outcomes. The Functional Resonance Analysis Method (FRAM) provides an appropriate approach for representing complex system processes by identifying system functions, functional variability, and interactions among operational activities [27,28]. However, FRAM primarily provides a qualitative description of functional structures and cannot directly assess system performance under uncertainty [29]. Therefore, dynamic assessment requires a probabilistic approach capable of quantifying the effects of functional variability on overall system performance. A Bayesian network (BN) provides advantages in probabilistic reasoning and uncertainty analysis and can dynamically assess system states, thereby complementing the limitations of FRAM [30,31]. Integrating FRAM with BN therefore provides a methodological pathway from representing complex functional interactions to quantitatively assessing surge capacity and identifying functional conditions that may constrain or support sustained emergency operations [32].

In summary, this study develops a resilience-oriented function–capability framework for assessing hospital surge capacity from a systems perspective. The framework aims to clarify how operational functions and their interactions shape the capabilities required to sustain essential healthcare services and emergency response under evolving disruptions. The hierarchical relationships among operational functions, capability dimensions, and overall surge performance is established. Specifically, the framework first represents the surge process in terms of operational functions and resilience-oriented system dimensions, then constructs a FRAM model to capture functional interactions and integrates it with BN to dynamically assess system performance under different emergency scenarios. This framework not only provides a theoretical foundation for understanding hospital surge processes and offers a more comprehensive explanation of how these processes generate different capability outcomes, but also provides a quantitative, model-based approach for assessing system performance under the specified scenario.

This study makes three contributions. First, it develops a function–capability framework that extends existing resource-oriented surge-capacity assessments by establishing a hierarchical representation linking operational functions to system capabilities. Second, it integrates improved FRAM with BN, combining functional modeling with probabilistic assessment to provide a comprehensive approach for evaluating emergency surge processes in complex medical systems. Third, the proposed framework provides an interpretable and structured approach for identifying critical functional elements and assessing surge capacity under different modeled disaster conditions, with potential applicability to other hospital emergency contexts.

## 2. Research Framework

Hospital surge capacity theory posits that a hospital’s ability to rapidly increase and sustain healthcare service provision during major emergencies fundamentally depends on the adaptive mobilization and reconfiguration of medical resources and organizational capabilities. Hick et al. were among the first to propose that hospital surge capacity should be enhanced at both the healthcare facility and community levels to increase the capacity to receive and treat patients [5]. Subsequently, Barbisch and Koenig further conceptualized hospital surge capacity in terms of four core components—Space, Staff, Stuff, and System—thereby establishing the basic framework of modern hospital surge capacity theory [33]. The Institute of Medicine, in its Crisis Standards of Care framework, further emphasized that hospital surge capacity involves not only the reconfiguration of internal hospital resources but also system-level coordinated expansion supported by regional healthcare systems and external support networks [34]. Accordingly, this study identifies System, Space, Staff, Stuff, and External Support as the fundamental components of hospital surge capacity, which collectively provide the resource foundation for maintaining essential healthcare services under prolonged, cascading, or compounding disruptions. This theory addresses the question of which resource and organizational elements enable hospitals to achieve surge expansion, thereby providing a theoretical basis for defining the resource composition of hospital surge systems and their capacity to sustain emergency service delivery under resource constraints.

However, a resource-expansion perspective alone remains insufficient to explain how surge resources are transformed into sustained emergency response capabilities, particularly when hospitals face prolonged, recurrent, or interacting disruptions. In recent years, hospital resilience theory has increasingly become an important theoretical foundation for research on emergency management in healthcare systems. Kruk et al. argued that, as complex adaptive systems, hospitals derive their emergency response capabilities not only from resource reserves but also from their ability to maintain healthcare services, adapt to environmental changes, and sustain recovery under disruptive conditions [35]. Blanchet et al. further proposed, from the perspectives of governance and capacity building, that hospital resilience emerges from continuous coordination among internal organizational functions and between the organization and its external environment [36]. Through a systematic review, Khalil et al. further identified key characteristics of hospital resilience, including robustness, adaptability, resourcefulness, continuity, and learning and transformation [37]. Their findings emphasize that hospital resilience is essentially a systemic capability generated through the continuous coupling, dynamic adjustment, and coordinated optimization of organizational functions. Hospital resilience theory therefore further addresses the question of how surge resources are transformed through organizational operations into sustained response capabilities, thereby providing theoretical support for explaining the formation mechanism of hospital surge capacity and its contribution to more resilient emergency healthcare systems.

Based on the two theoretical perspectives discussed above, this study argues that hospital surge capacity is not a direct consequence of a simple increase in resource quantity, but rather a comprehensive capability generated through the combined effects of surge resources and organizational resilience, enabling hospitals not only to absorb immediate shocks but also to adapt and maintain essential functions under evolving disruptions. Hospital surge capacity theory provides the resource foundation for capability generation, whereas hospital resilience theory elucidates the operational mechanism through which these resources are reconfigured, coordinated, and sustained over time. These two theories together constitute the theoretical basis for analyzing hospital surge capacity as an adaptive system capability that connects immediate emergency response with the sustained provision of healthcare services. To operationalize the integration of these two theoretical perspectives, the five capability dimensions used in this study were established a priori through a theory-driven synthesis. The derivation followed a two-stage mapping logic. First, each capability dimension was anchored in one or more hospital surge-capacity components that represent its underlying resource and organizational foundation. Second, these components were aligned with corresponding resilience attributes that characterize how resources are reconfigured, mobilized, sustained, coordinated, and improved under disruptive conditions. On this basis, five capability dimensions were defined: functional reconfiguration capability, resource configuration capability, operational continuity capability, regional coordination capability, and collaborative optimization capability. FRAM was subsequently used to identify and assign operational functions to these theoretically predefined capability dimensions under different surge configurations. Thus, the theoretical synthesis defines the capability categories, whereas the expert-informed functional analysis provides their operational representation.

**Figure 1:**
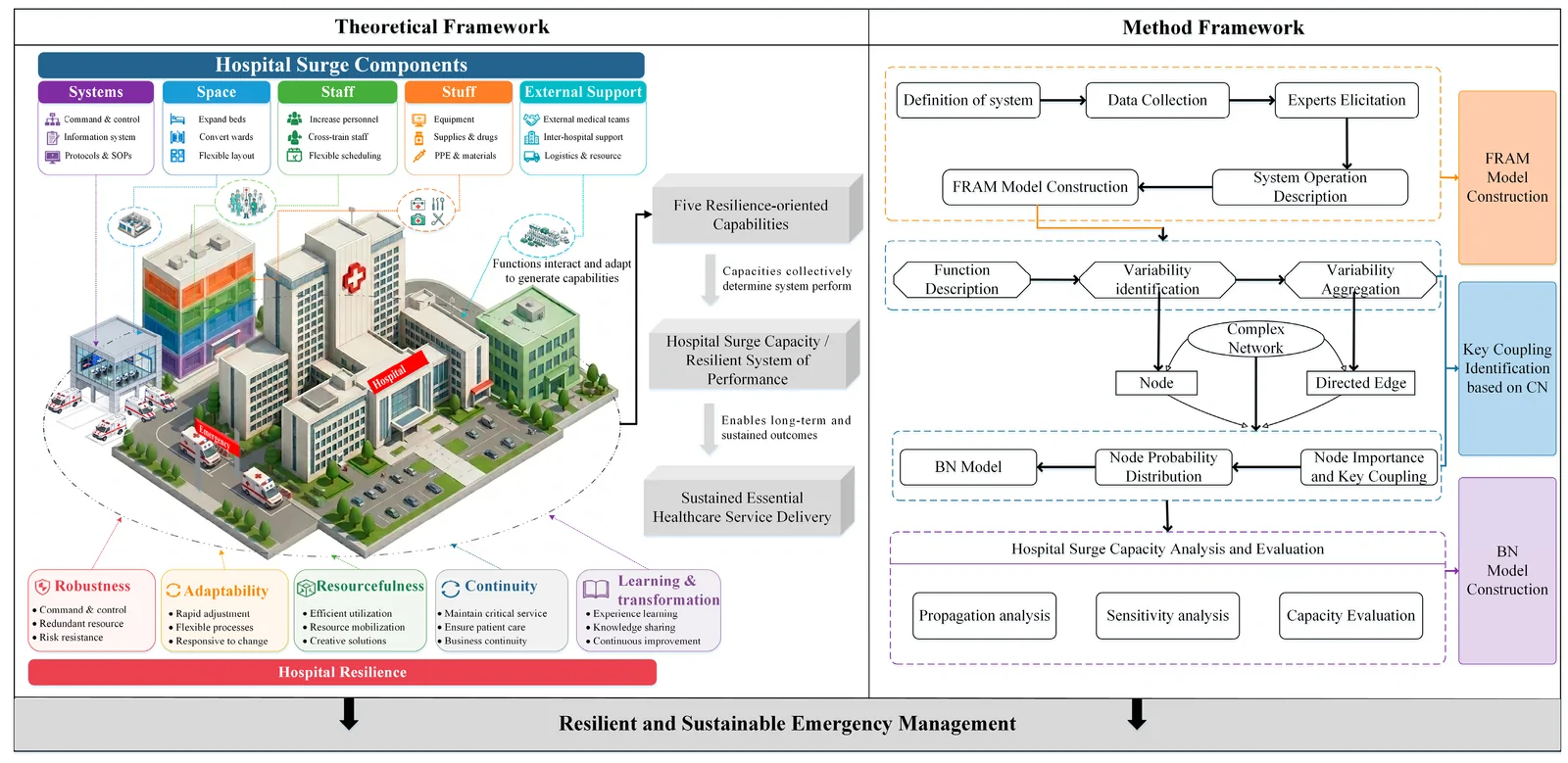
Research theory and method framework.

In terms of the methodological framework, this study first defines the system, collects relevant data, and conducts expert consultations to clarify the research boundaries, operational processes, and principal functional activities of the hospital surge system, on the basis of which a FRAM model is developed. Through function description, identification of functional variability, and variability aggregation, the model characterizes the functional states and interdependencies involved in ward conversion, personnel deployment, supply support, clinical treatment, information transmission, and command decision-making. On this basis, the functions and their coupling relationships are transformed into a complex network composed of nodes and directed edges, and an improved K-shell method is employed to identify critical functions and critical couplings. The complex network analysis results are then incorporated into a BN model, in which node importance is used to determine node probability distributions and probabilistic dependencies among nodes are established. The resulting methodological framework therefore forms an analytical chain of “FRAM functional modeling, identification of critical couplings, and BN capability inference,” allowing structural vulnerabilities and capability dependencies to be assessed under different emergency configurations and uncertain operating conditions. By integrating these theoretical and methodological approaches, this study can not only identify the functions that constitute the hospital surge system and explain how these functions are coupled, but also clarify how critical functions generate surge capacity and how variations in different functions affect the overall level of surge capacity. More importantly, the framework shifts hospital surge assessment from a short-term resource-expansion perspective toward an adaptive capability perspective, providing a basis for maintaining essential healthcare services and supporting more resilient emergency management under complex and potentially compounding risks. The theoretical and methodological framework of the study is shown in Figure 1.

## 3. Research Method

### 3.1. Construction of Improved FRAM Model

**Figure 2:**
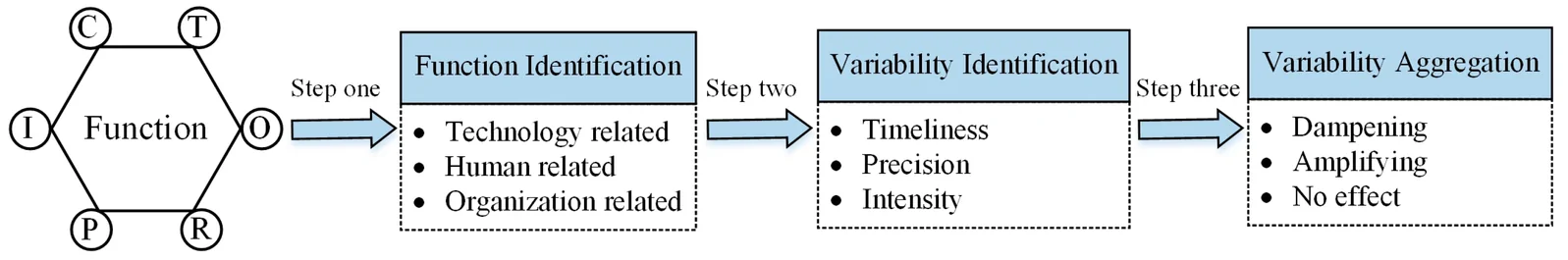
Modeling process of FRAM.

The FRAM is designed to qualitatively investigate the principles underlying the successful functioning of complex sociotechnical systems. Following the approach outlined by Hollnagel [27], this study employs FRAM to elucidate the functions involved in the systematic operation of a hospital surge system for responding to emergencies. A fundamental task in conducting a FRAM analysis is to identify the functions involved in the system, which is regarded as the starting point of the analysis. Each function within the target system is graphically represented as a hexagon comprising six aspects: input (I), output (O), preconditions (P), resources (R), control (C), and time (T). Following function identification, functional variability must be sequentially identified, aggregated, and managed to construct the FRAM model, as illustrated in Figure 2.

#### 3.1.1. Data Collection and Function Identification

In complex sociotechnical systems, system functions emerge from the interactions among technological, human, and organizational elements. This complex relationship makes it difficult to describe accurately, and it is also quite challenging to quantitatively evaluate six aspects of each function. The first step in constructing the FRAM model is to identify and describe the relevant system functions. The second step is to develop scenarios involving sudden increases in medical demand and to identify the relationships and variability among functions under different scenarios. Expert interview is widely recognized as an effective method for identifying and describing system functions. Accordingly, two rounds of expert interviews were conducted in this study [38]. The experts recruited for this study were primarily from fields related to hospital surge capacity, including hospital management, medical emergency, and healthcare. Expert selection was based mainly on relevant professional backgrounds, more than 10 years of work experience, and practical experience in responding to major public health emergencies or managing hospital emergency operations. Their expertise was used to assess the functions involved in hospital surge processes and the relationships among these functions from the professional perspective of EMS. A total of 25 experts from 3 tertiary general hospitals and 2 medical institutions in China participated in the interviews. Specifically, 5 experts were from Hospital A, 5 from Hospital B, 5 from Hospital C, 5 from the Emergency Medical Rescue Center, and 5 from the municipal medical emergency response team. Both rounds of interviews were conducted using a semi-structured interview approach, with the 25 experts participating in the respective rounds according to the consultation topics. Each interview lasted approximately 60–90 min. An initial interview guide was developed based on the research questions and subsequently revised following expert review. Before each interview, the purpose of the study was explained to the experts, and informed consent was obtained. The basic characteristics of the experts are presented in Table 1, and the interview guide is provided in Table 2. The interviews were conducted as professional consultations to obtain expert judgments on hospital surge operations. They did not involve patients or the collection of personal, sensitive, or patient-related information, and informed consent was obtained from all participating experts.

The first round of interviews was primarily conducted to identify the functions involved in the hospital surge process. Based on the data obtained from the expert consultation and following the basic principles of the Structured Analysis and Design Technique (SADT), this study analyzed the hospital surge process from three dimensions: personnel, technology, and organization. First, the experts reviewed the hospital surge process in the context of major public health emergencies based on their professional knowledge and practical experience in emergency response, identifying the major activities involved and their functional requirements. Second, based on the experts’ judgments, the core functions involved in the process were identified, discussed, and confirmed, with their definitions and boundaries further refined. Third, once the functions had been established, the experts further discussed the six aspects of FRAM associated with each function. This process ultimately resulted in the identification of 24 basic functions and their corresponding six FRAM aspects. It should be noted that not all six aspects are necessarily assigned to every function, although upstream functions constitute fundamental components of the analysis and must involve at least an input and an output.

The second round of interviews was primarily conducted to construct representative scenarios of sudden increases in medical demand at hospitals and to further identify the relationships and variability among functions under different scenarios. Based on the 24 basic functions identified in the first round, the second round focused on qualitative data and information regarding resource organization, functional adjustments, and operational coordination during hospitals’ emergency responses to sudden incidents. Drawing on the experts’ institutional backgrounds and practical experience in emergency response, the interviews further explored how hospitals organize and operate under different sudden medical demand scenarios and constructed several representative hospital surge scenarios. These scenarios do not represent any single hospital; rather, they were developed by synthesizing common organizational practices and operational characteristics identified across the participating institutions. By comparing and analyzing the resource organization and operational coordination approaches that repeatedly emerged and differed substantially in the actual emergency response processes of the participating hospitals, four representative hospital surge scenarios were identified from the interview data. For these four scenarios, corresponding assumptions were made regarding the form of resource expansion (local use vs. cross-regional support) and the mode of resource organization (unitization vs. modularization) during the hospital surge process. The first scenario (*S*_1_) assumes that hospitals primarily rely on existing internal resources and achieve surge expansion through the adjustment and conversion of medical functional modules. The second scenario (*S*_2_) also assumes reliance on internal resources, but further divides resources and functions into relatively independent operational units to maintain closed-loop operations within each unit. The third scenario (*S*_3_) assumes the use of external resources, with specialized medical teams deployed to provide targeted, modular support for specific tasks or geographic areas. The fourth scenario (*S*_4_) also assumes the use of external resources, but involves the deployment of an entire medical formation into the emergency response system, thereby providing comprehensive medical capabilities. The four scenarios for hospital surge capacity are shown in Table 3. In this study, they were used as representative operational scenarios for FRAM modeling and comparative capability assessment rather than as an exhaustive classification of hospital surge arrangements. After the representative scenarios were established, the second round of interviews further examined the interrelationships among functions and their variability across different scenarios. Following the principles for identifying functional variability, the six aspects of each identified function were described and compared to further derive the coupling relationships among functions and their variations across scenarios. Through expert discussions on different combinations, couplings, and variabilities of functions, the typical operational scenarios of hospital surge and the corresponding configurations of functional relationships were ultimately established. The functional variability was limited to variability in the couplings between upstream and downstream functions, and selected results are presented in Table 4. The above interview results provide a basis for FRAM-BN modeling.

#### 3.1.2. Variability Identification and Aggregation

The second step in constructing the FRAM model is to identify functional variability. Functional variability can theoretically be classified into potential variability and actual variability, and the latter is adopted in this study for empirical analysis. In practice, functional variability is characterized by variability in the output of the target function. Therefore, examining the question of why a function’s output varies helps identify the sources of functional variability. Considering the scenario evolution and functional characteristics of hospital emergency response, functional variability in this study is assessed in terms of timeliness, precision, and intensity. Timeliness $V_{\mathrm{aj}}^{T}$ is classified into 4 states: on time, early, late, and no effect. Precision $V_{\mathrm{aj}}^{P}$ is classified into 4 states: precise, acceptable, imprecise, and wrong. Intensity $V_{\mathrm{aj}}^{I}$ is classified into 3 states: normal, strong, and weak. The ranking and scoring of these categories were determined based on a review of the relevant literature and interviews with experts who provided professional judgments. These three dimensions were then used to characterize the variability in the output *a* of each function *j*.

**Figure 3:**
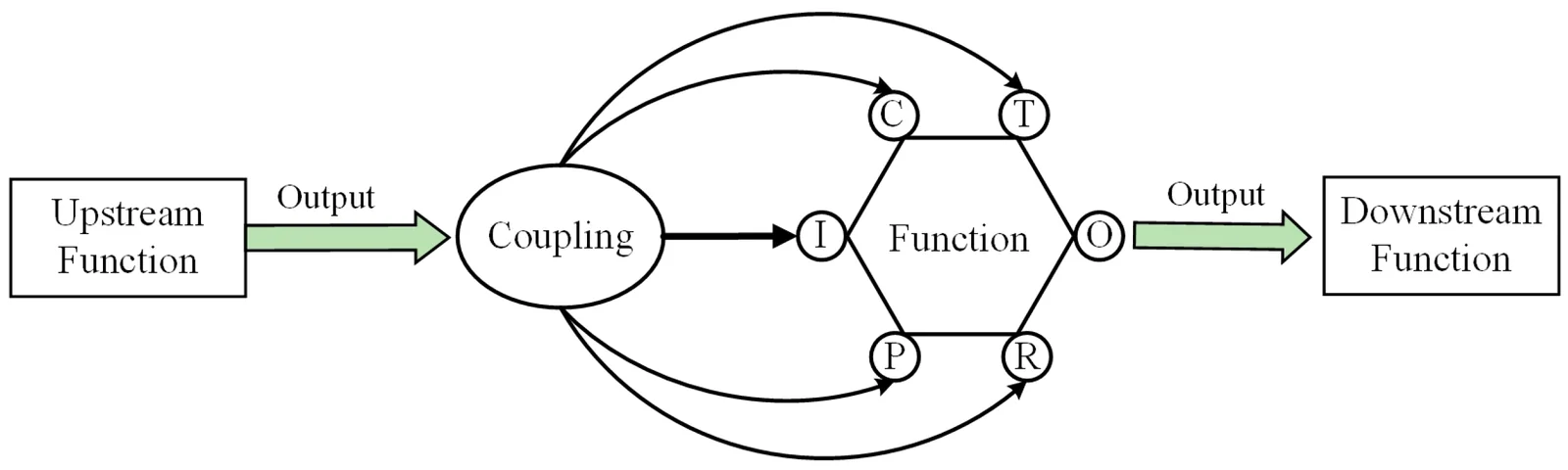
Aggregation diagram of functional variability.

In addition to functional variability, this study considers coupling variability, which describes how functional variability propagates between interconnected functions. Different functions are interconnected through couplings among the identified functions, thereby giving rise to the process of functional resonance [27]. In the FRAM model, coupling is also referred to as “upstream–downstream coupling” whereby the output variability of an upstream function may influence the subsequent performance of a downstream function. Coupling effects are classified as dampening, amplifying, or no effect. A dampening effect indicates that upstream functional variability is attenuated as it propagates to the downstream function, whereas an amplifying effect indicates that the variability is strengthened during propagation. No effect indicates that the variability is neither substantially attenuated nor amplified during transmission. These categories characterize the propagation magnitude of functional variability rather than a direct effect or risk evaluation. As shown in Figure 3, the output of an upstream function may serve as one of the five aspects of the target function, after which the resulting coupling affects the downstream function through the output of the target function. Accordingly, the coupling variability associated with timeliness, precision, and intensity is denoted by $CV_{\mathrm{ija}}^{T}$, $CV_{\mathrm{ija}}^{P}$, and $CV_{\mathrm{ija}}^{I}$, respectively.

Finally, based on the evolution of the emergency response process to public health emergencies, variability arising from different working conditions under different scenarios was examined and denoted as $V_{\mathrm{aj}}^{S}$. The scenarios considered three categories of working conditions: organizational factors, human factors, and disaster-related factors. Organizational factors included the provision of medical resources, staffing of healthcare personnel, training levels, and task complexity. Human factors included workload, fatigue, and the physical and psychological condition of individuals. Disaster-related factors referred to the possibility that increasing disaster severity during scenario evolution could disrupt the normal operation of the system. In summary, the variability index VPN is expressed in Equation (1) [39]:

(1) $$\mathrm{VPN}=V_{\mathrm{aj}}^{T}\times V_{\mathrm{aj}}^{P\;\mathrm{or}\;I}\times CV_{\mathrm{ija}}^{T}\times CV_{\mathrm{ija}}^{P\;\mathrm{or}\;I}\times V_{\mathrm{aj}}^{S}$$

where the $V_{\mathrm{aj}}^{T}$ denotes the timeliness variability of output *a* of function *j*; $V_{\mathrm{aj}}^{P\;\mathrm{or}\;I}$ denotes the precision or intensity variability of output *a* of function *j*; $CV_{\mathrm{ija}}^{T}$ is intensity denotes the coupling variability in timeliness associated with the transmission of output *a* from function *i* to *j*; $CV_{\mathrm{ija}}^{P\;\mathrm{or}\;I}$ denotes the corresponding coupling variability in precision or intensity; and $V_{\mathrm{aj}}^{S}$ denotes the variability arising from working conditions for output *a* of function *j* under scenario *s*, as expressed as Equation (2). Here, *i* and *j* represent the upstream and downstream functions, respectively, a denotes the output of the function, and *s* denotes the scenario.

(2) $$V_{\mathrm{aj}}^{S}=\max\left\{1,\log\left(\sum_{k=1}^{n}W_{s}^{n}\times V_{\mathrm{ija}}^{n}\right)\right\}$$

where $W_{s}^{n}$ is variability of working conditions under scenario *s* and $V_{\mathrm{ija}}^{n}$ is effect of working conditions on output *a* of function *j*.

The ranking and numerical coding of the variability categories were established through a structured review of the relevant documents and methodological literature and were subsequently examined during expert interviews. Experts identified the applicable variability states and coupling effect categories for each function and specified the corresponding value. Table 5 summarizes the quantitative scoring criteria for the variability-related parameters, and entered into the VPN calculation. For example, if an upstream output was assessed as “early” ($V_{\mathrm{aj}}^{T}$ = 2) with an amplifying coupling effect ($CV_{\mathrm{ija}}^{T}$ = 2), while its precision was assessed as “acceptable” ($V_{\mathrm{aj}}^{P}$ = 2) with no coupling effect ($CV_{\mathrm{ija}}^{P}$ = 1), the corresponding combined variability score was 2×2×2×1 = 8. Initial judgments were elicited separately from experts during the interview process. Where judgments differed, the research team rechecked the alternative assessments against available operational records, emergency reports, and relevant documentary evidence, after which discrepant items were revisited through follow-up discussion. Final parameter specifications were determined through an evidence-informed consensus procedure.

Probability distributions were assigned to the variables described above through expert interviews and repeated discussions, and a Monte Carlo simulation (MCS) was employed to calculate VPN and determine coupling variability. Different scenarios were constructed by combining the four stages of emergency management during disaster evolution—prevention, preparedness, response, and recovery—with varying organizational and personnel conditions. A total of 1000 iterations were performed for each coupling, and the VPN of each coupling was subsequently determined. The resulting VPN values were then compared with a predefined threshold, and a coupling was regarded as effective when its VPN was equal to or greater than 6 in more than 5% of all simulation iterations [40]. This approach effectively overcomes the limitations of relying solely on experiential judgment to assess coupling variability. Moreover, modeling human and organizational functions using precisely assigned numerical values can compensate for insufficient information, ambiguity, and the uncertainty inherent in real-world decision-making conditions. This type of variability identification has gained increasing recognition and has demonstrated greater sensitivity [41].

### 3.2. BN Modeling and Inference

The traditional FRAM approach can effectively characterize functional composition, interactions, and performance variability in complex systems, but it does not directly quantify how uncertainty in individual functional performance propagates to higher-level system capabilities. Therefore, BN is introduced to represent and propagate performance uncertainty from the functional level to the capability levels.

#### 3.2.1. Principle of BN

As a probabilistic graphical model, BN have been widely applied to address complexity and uncertainty in risk analysis [42]. A BN represents a set of variables and their conditional dependencies through a directed acyclic graph. The joint probability distribution of all model variables can be factorized according to their local dependency structure, as expressed in Equation (3) [43]:

(3) $$p(Z)=\prod_{i=1}^{n}p\left(Z_{i}|P_{a}(Z_{i})\right)$$

where **Z** = {*Z*_1_, …, *Z_n_*} denotes the set of model variables and *P_a_*(*Z_i_*) represents the parent set of node *Z_i_*.

In this study, the root nodes are modeled as continuous stochastic functional-performance variables, whereas the capability nodes and the top-level surge-capacity node are represented by continuous conditional relationships determined by their parent nodes. Accordingly, the BN is interpreted as an uncertainty propagation framework.

#### 3.2.2. Improved K-Shell Algorithm for Structural Importance

An improved K-shell decomposition algorithm was employed to calculate the structural importance of functional nodes, which was subsequently used to determine their relative contribution weights in capability aggregation. To quantify the structural contribution, the FRAM model is transformed into a directed weighted network *G* = (*V*,*E*), where *V* = {*v*_1_, …, *v_n_*} denotes the set of functional nodes and *E* = {*e*_1_, …, *e_m_*} denotes the set of directed functional couplings [44]. The corresponding weighted adjacency matrix *A* is defined as shown in Equation (4):

(4) $$A_{\mathrm{ij}}=\left\{\begin{array}{ll} a_{\mathrm{ij}}\omega_{\mathrm{ij}}, & v_{i}\to v_{j}\\ 0, & \mathrm{otherwise} \end{array}\right.$$

where *a_ij_* indicates whether a directed coupling exists from functions *i* to *j*, and *ω_ij_* denotes its coupling weight.

The structural importance of node *i* is evaluated jointly from its degree and weighted connectivity. The total degree *D_i_* and cross degree *CD_i_* of node *i* are calculated as shown in Equation (5):

(5) $$D_{i}=d_{i}^{\mathrm{in}}+d_{i}^{\mathrm{out}}=\sum_{j\in V}a_{\mathrm{ji}}+\sum_{j\in V}a_{\mathrm{ij}},\;CD_{i}=d_{i}^{\mathrm{in}}d_{i}^{\mathrm{out}}=\sum_{j\in V}a_{\mathrm{ji}}\sum_{j\in V}a_{\mathrm{ij}}$$

where $d_{i}^{\mathrm{in}}$ and $d_{i}^{\mathrm{out}}$ denote the in-degree and out-degree of node *i*, respectively.

The weighted connectivity of node i is further characterized by its total strength *W_i_* and cross strength *CW_i_*, which are calculated using Equation (6):

(6) $$W_{i}=W_{i}^{\mathrm{in}}+W_{i}^{\mathrm{out}}=\sum_{j\in V}\left(\omega_{\mathrm{ji}}+\omega_{\mathrm{ij}}\right),\;CW_{i}=W_{i}^{\mathrm{in}}W_{i}^{\mathrm{out}}=\sum_{j\in V}\omega_{\mathrm{ji}}\sum_{j\in V}\omega_{\mathrm{ij}}$$

where $W_{i}^{\mathrm{in}}$ and $W_{i}^{\mathrm{out}}$ denote the in-strength and out-strength of node *i*, respectively.

The degree-based and strength-based connectivity measures are then integrated to obtain the initial structural-importance index *ψ_i_*, as expressed in Equation (7):

*ψ_i_* = (*D_i_* + *CD_i_*)(*W_i_* + *CW_i_*) (7)

This index is incorporated into the K-shell decomposition to distinguish nodes with different positions and connectivity patterns. After accounting for the iterative ordering of nodes within the same shell, the final structure-informed importance of function *i* is obtained using Equation (8):

(8) $$\psi_{i}^{\mathrm{Ki}}=\psi_{i}^{K}+\Gamma_{i}^{\xi K}$$

where $\psi_{i}^{K}$ denotes the shell-level structural importance and $\Gamma_{i}^{\xi K}$ is the iteration factor used to further differentiate nodes within the same shell [45].

The resulting $\psi_{i}^{\mathrm{Ki}}$ represents the relative structural importance of function *i* in the network. It is used subsequently to determine the relative contribution weights of functions in capability generation and is not interpreted as a probability or as a parameter of the functional-performance distribution.

#### 3.2.3. Functional-Performance Distribution and Propagation

The root nodes of the BN represent the uncertain performance of individual functions rather than the probability of function occurrence or failure. To characterize this uncertainty, the variability scores obtained for each function under different scenarios were transformed into bounded functional-performance values. Because larger variability scores indicate less desirable functional states, the variability score of function *i* for dimension *d* in the *r*th Monte Carlo iteration is transformed into a normalized functional-performance value $x_{\mathrm{id}}^{r}$ using Equation (9):

(9) $$x_{\mathrm{id}}^{r}=1-\frac{z_{\mathrm{id}}^{r}-z_{d}^{\min}}{z_{d}^{\max}-z_{d}^{\min}}$$

where $z_{\mathrm{id}}^{r}$ denotes the sampled variability score of function *i* for dimension *d*, and $z_{d}^{\min}$ and $z_{d}^{\max}$ denote the lower and upper bounds of the corresponding scoring scale. Consequently, $x_{\mathrm{id}}^{r}\in \left[0,1\right]$, with a higher value indicating better functional performance.

For each function, the normalized scores of the two selected output-variability dimensions were aggregated with equal weights to obtain its performance value in the *r*th simulation, as defined in Equation (10):

(10) $$X_{i}^{r}=\frac{x_{\mathrm{iT}}^{r}+x_{\mathrm{iP}}^{r}(\mathrm{or}\;x_{\mathrm{iI}}^{r})}{2}$$

where $X_{i}^{r}\in \left[0,1\right]$ represents the functional-performance level of node *i*. Repeated Monte Carlo sampling therefore generated an empirical performance distribution $\left\{X_{i}^{1},X_{i}^{2},\cdots ,X_{i}^{M}\right\}$ for each function. The sample mean and standard variance of the functional-performance observations are then calculated using Equation (11):

(11) $$\mu_{i}=\frac{1}{M}\sum_{r=1}^{M}X_{i}^{r},\;\;\;\;s_{i}=\sqrt{\frac{1}{M-1}\sum_{r=1}^{M}{\left(X_{i}^{r}-\mu_{i}\right)}^{2}}.$$

where *M* denotes the number of Monte Carlo iterations, *μ_i_* denotes the sample mean of the functional-performance observations for function *i*, and *s_i_* denotes the corresponding standard variance.

Because of *X_i_* ∈ [0, 1], a truncated-normal distribution was used as a bounded parametric approximation of the empirical distribution, as specified in Equation (12):

(12) $$X_{i}\sim \mathrm{TN}\left(\mu_{i},s_{i};0,1\right)$$

where the truncated-normal (*TN*) specification was adopted to provide a smooth and consistent representation of root-node uncertainty across functions and scenarios. Its adequacy was evaluated against the original empirical distributions. Across the 84 active function scenario combinations, the Wasserstein distance ranged from 0.044 to 0.060, with a median of 0.050, indicating limited distributional displacement on the [0, 1] scale. Because the empirical distributions contain discrete mass points arising from the ordinal variability scores, exact distributional equivalence was not expected.

The intermediate and the top-level node are modeled as continuous conditional nodes. Conditional on a given realization of their parent nodes, these relationships are deterministic. Because the parent functional-performance variables are stochastic, uncertainty is propagated through the network and induces probability distributions for each capability dimension and for overall hospital surge capacity. Formally, the conditional relationship for capability *C_k_* can be represented as Equation (13):

*p*(*C_k_*∣**X***_k_*) = *δ*[*C_k_* − *g_k_*(**X***_k_*)] (13)

where **X**_k_ denotes the set of functional-performance variables associated with capability *C_k_* and *δ*(·) represents the deterministic conditional relationship. The same principle applies to the top-level node. Therefore, the resulting distributions of the capability and overall-capacity nodes are induced by uncertainty propagation through the BN.

### 3.3. Measurement of Hospital Surge Capacity

**Figure 4:**
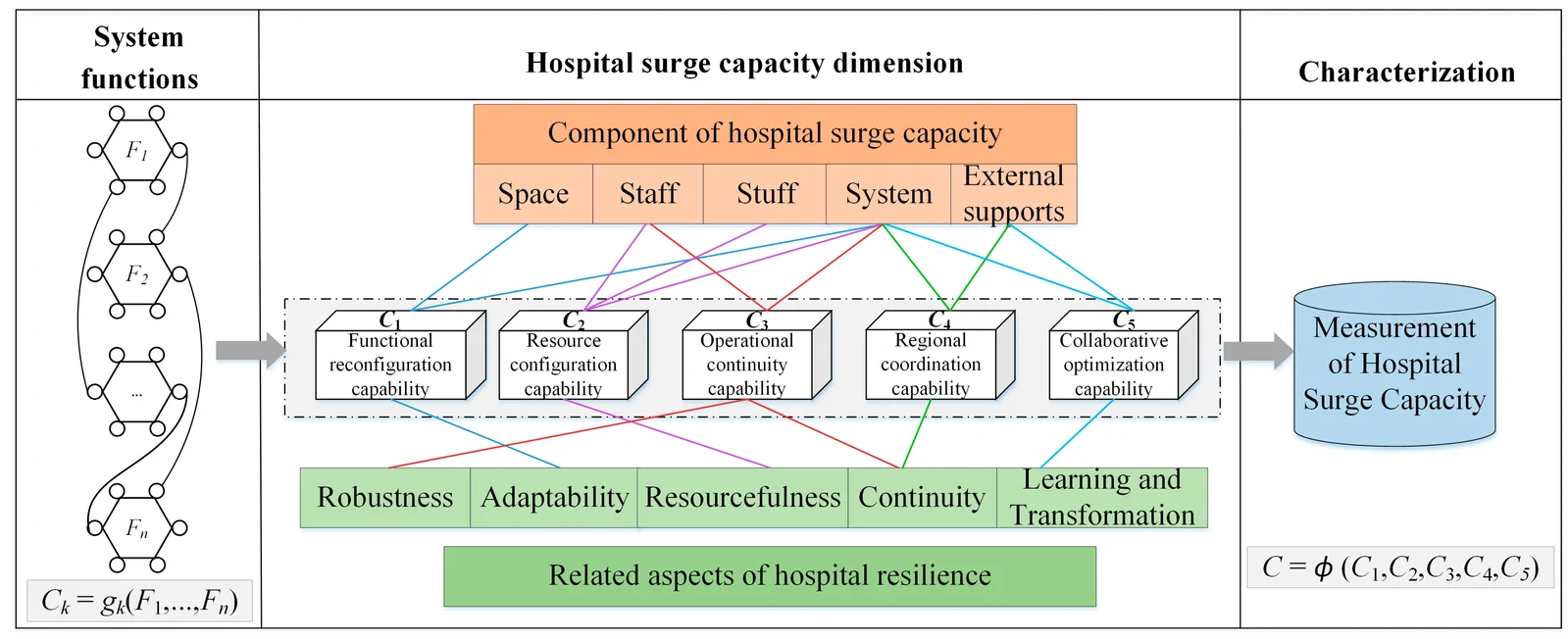
Theory driven function–capability framework for hospital surge-capacity assessment [5,35].

Based on the theory-driven mapping described in Section 2, hospital surge capacity is represented by five capability dimensions: functional reconfiguration capability (*C*_1_), resource configuration capability (*C*_2_), operational continuity capability (*C*_3_), regional coordination capability (*C*_4_), and collaborative optimization capability (*C*_5_). These dimensions were theoretically specified. Figure 4 shows the theoretical derivation of the five capability dimensions by linking the hospital surge-capacity components, resilience attributes, and operational functions. The specific FRAM functions assigned to each capability vary across surge configurations because different organizational arrangements activate different functional pathways.

(1) Functional reconfiguration capability (*C*_1_).

Functional reconfiguration capability refers to the hospital’s ability to rapidly reorganize medical functions, treatment processes, and physical spaces in response to changing emergency demands. It is theoretically anchored in the Space and System components of hospital surge capacity and primarily reflects the Adaptability attribute of hospital resilience. This capability enables hospitals to transform ordinary wards into intensive care units, redesign clinical workflows, and rapidly adjust organizational structures to support surge operations.

(2) Resource configuration capability (*C*_2_).

Resource configuration capability describes the hospital’s ability to dynamically mobilize, allocate, and optimize critical medical resources, including healthcare personnel, medical equipment, beds, and protective supplies. It originates mainly from the staff, stuff, and system dimensions of surge capacity and corresponds to the resourcefulness aspect of hospital resilience. This capability emphasizes efficient resource integration rather than resource quantity alone.

(3) Operational continuity capability (*C*_3_).

Operational continuity capability represents the hospital’s ability to maintain stable and continuous healthcare services under surge conditions while ensuring the uninterrupted operation of critical medical processes. This capability is supported by organizational systems, medical personnel, and essential resources, and is closely associated with the resilience characteristics of robustness and continuity. It reflects the hospital’s capacity to sustain core medical functions despite substantial operational pressure.

(4) Regional coordination capability (*C*_4_).

Regional coordination capability refers to the hospital’s ability to coordinate patient transfers, resource sharing, and collaborative medical responses across multiple healthcare institutions. It is primarily supported by external support and system-level coordination within the surge capacity framework and is closely related to the resilience characteristics of continuity and resourcefulness. This capability facilitates regional healthcare integration and improves the overall efficiency of emergency medical networks.

(5) Collaborative optimization capability (*C*_5_).

Collaborative optimization capability reflects the hospital’s ability to continuously improve emergency response performance through information sharing, cross-organizational collaboration, dynamic decision-making, and organizational learning. It mainly derives from the system and external support components of surge capacity and corresponds to the learning and transformation dimension of hospital resilience. Rather than representing a specific expansion mode, this capability emphasizes continuous optimization of organizational coordination and adaptive governance throughout the emergency response process.

After establishing the five capability dimensions, the operational functions identified through FRAM are assigned to the corresponding capability dimensions according to the theory-driven mapping described above. Because different surge configurations activate different functional pathways, the set of functions contributing to each capability may vary across scenarios. Let $R_{k}^{s}\subseteq F^{s}$ denote the set of functional nodes assigned to capability *C_k_* under scenario *s*, where *k* = 1, …, 5, and let $X_{i}^{s}$ denote the continuous functional-performance random variable of function *i* defined in Section 3.2.3. To avoid double counting, each active function is assigned to one capability dimension only within a given scenario.

The use of K-shell importance as aggregation weights is based on the structural role of functions in the FRAM coupling network. In a functionally coupled system, a function that is more deeply embedded in the network core and connected through stronger and more extensive coupling pathways has greater structural potential to participate in the generation and propagation of system capability [45]. Accordingly, the normalized K-shell importance is used as a structure-informed measure of relative contribution within the aggregation model. For function *i* belonging to capability *C_k_*, its within-capability weight is defined as Equation (14):

(14) $$w_{\mathrm{ik}}^{s}=\frac{\psi_{i}^{s}}{\sum_{j\in R_{k}^{s}}\psi_{j}^{s}}$$

where $\psi_{i}^{s}$ denotes the structural importance of function *i* under scenario *s* and $R_{k}^{s}\subseteq F^{s}$ denotes the set of functional nodes assigned to capability *C_k_* under scenario *s*.

The performance of Capability *C_k_* is obtained by aggregating the functional-performance variables assigned to that capability according to their within-capability structural weights, as expressed in Equation (15):

(15) $$C_{k}^{s}=g_{k}^{s}(X)=\sum_{i\in R_{k}^{s}}w_{\mathrm{ik}}^{s}X_{i}^{s},\;\;\;\;k=1,\ldots ,5$$

where $C_{k}^{s}$ denotes the performance of capability *C_k_* under scenario s, $X_{i}^{s}$ denotes the functional-performance variable of function *i*, and $w_{\mathrm{ik}}^{s}$ denotes its within-capability weight. $C_{k}^{s}$ is a stochastic performance variable whose distribution is induced by uncertainty propagation from its constituent functional-performance variables, and $X_{i}^{s}$ is also a stochastic performance variable. The functions *g_k_*(·) represent scenario-specific aggregation of stochastic functional performance.

The relative structural weight of each capability dimension in overall hospital surge capacity is determined by the aggregate structural importance of the functions assigned to that capability. The aggregate structural importance $I_{k}^{s}$ of capability $C_{k}^{s}$ under scenario *s* is calculated as shown in Equation (16):

(16) $$I_{k}^{s}=\sum_{i\in R_{k}^{s}}\psi_{i}^{s}$$

The aggregate capability importance is then normalized across the five capability dimensions to obtain the $\eta_{k}^{s}$, as defined in Equation (17):

(17) $$\eta_{k}^{s}=\frac{I_{k}^{s}}{\sum_{h=1}^{5}I_{h}^{s}}$$

where $\eta_{k}^{s}$ denotes the normalized structural weight of capability *C_k_* under scenario *s*.

Finally, overall hospital surge capacity *C^s^* is expressed in Equation (18):

(18) $$C^{s}=\phi^{s}(C_{1},\ldots ,C_{5})=\sum_{k=1}^{5}\eta_{k}^{s}C_{k}^{s},\;\sum_{i\in R_{k}^{s}}w_{\mathrm{ik}}^{\left(s\right)}=1,\;\sum_{k=1}^{5}\eta_{k}^{s}=1$$

where *ϕ*(·) integrates the five capability dimensions according to their relative structural importance. The five capability weights sum to 1. Because functional-performance variables are stochastic, their uncertainty propagates through the BN to the capability nodes and the overall hospital surge-capacity node. Thus, both capability level and overall surge-capacity performance are represented as continuous variables.

## 4. Case Analysis

### 4.1. FRAM Model of Hospital Surge

**Figure 5:**
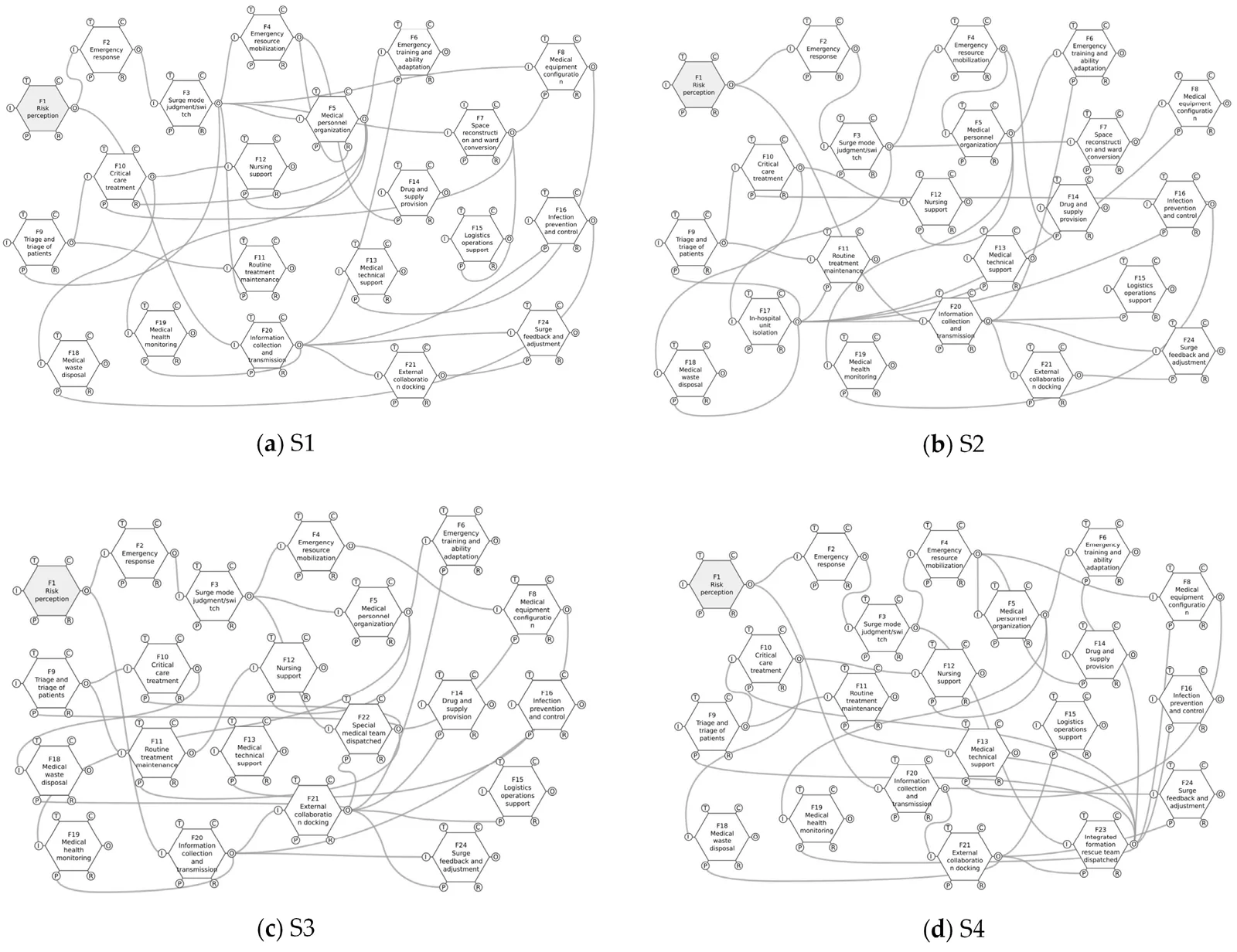
FRAM model of hospital surge under different scenarios.

Following the principles of FRAM, the functional descriptions collected in this step and the couplings listed in Table 4 are integrated into the FRAM Model Visualizer to construct the hospital surge FRAM model, as shown in Figure 5.

### 4.2. Functional Characterization of Hospital Surge Capacity

Based on the five capability dimensions, the identified functions were assigned to the corresponding capability dimensions under each surge scenario. Because different organizational configurations activate different functional pathways, the functional composition of each capability varies across scenarios. The resulting function–capability mappings are presented in Table 6.

### 4.3. Network Structural Importance and Functional-Performance Modeling

#### 4.3.1. Network Topology Analysis

**Figure 6:**
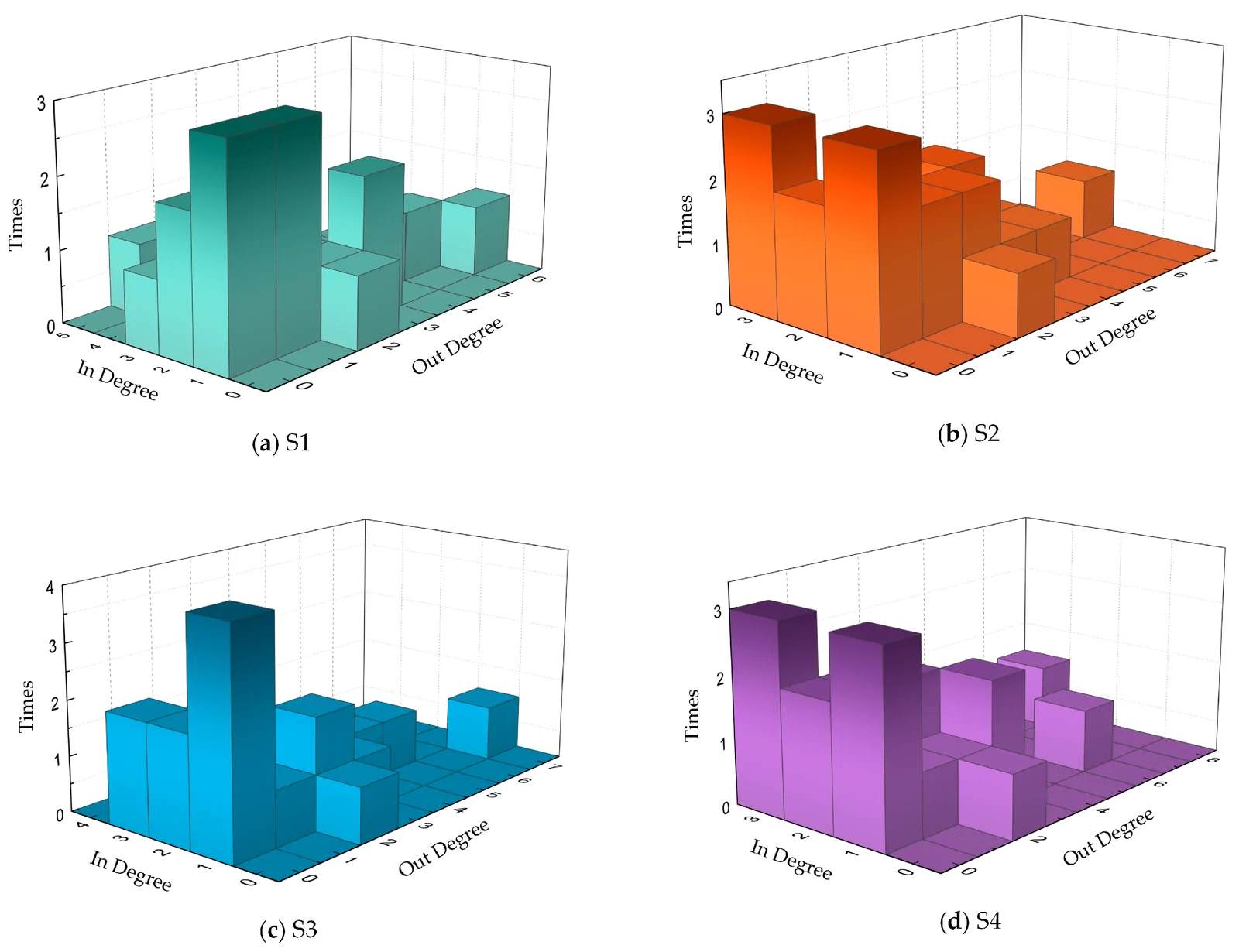
Node degree distribution under different scenarios.

**Figure 7:**
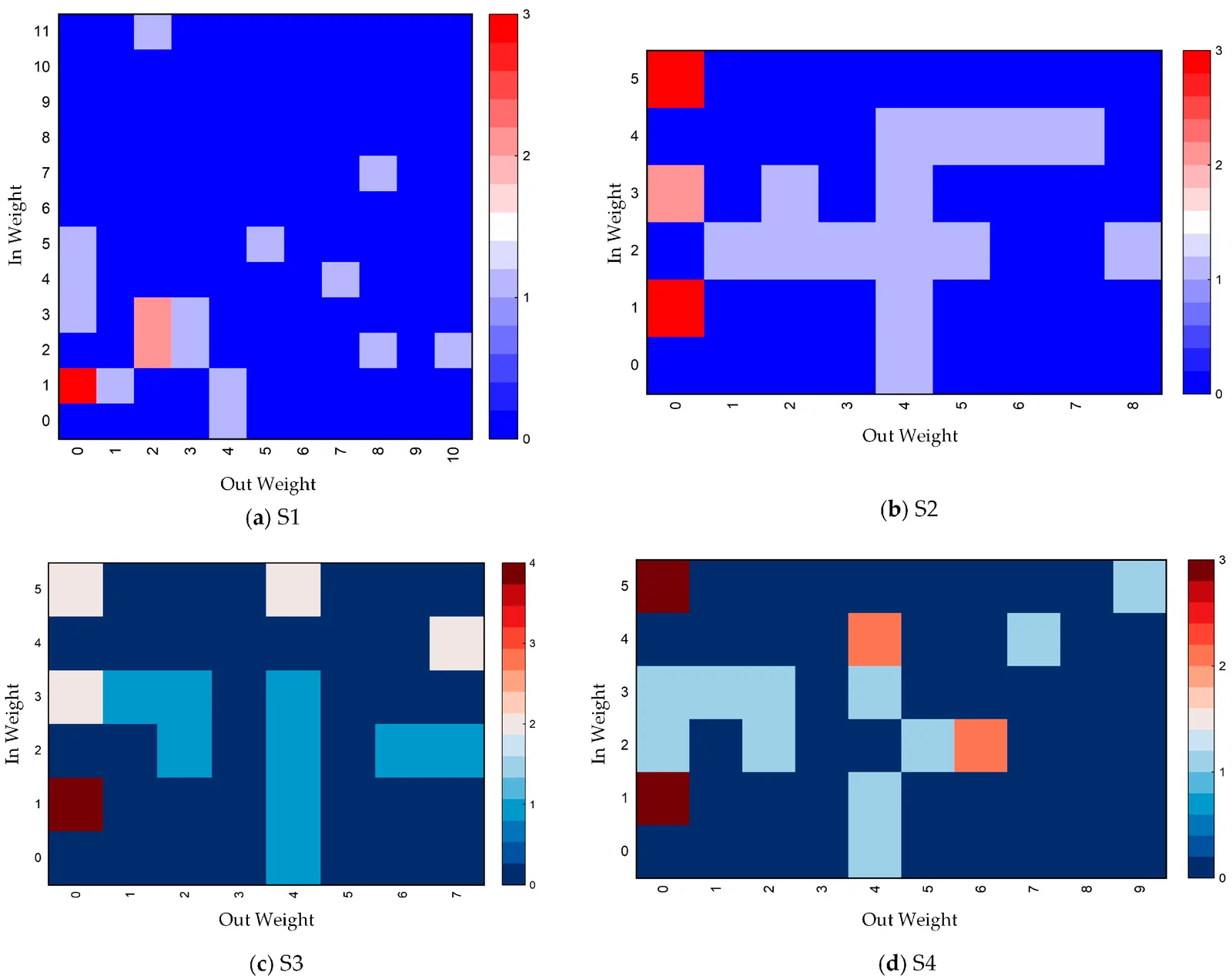
Edge-weight distribution under different scenarios.

Based on the FRAM models shown in Figure 5, the functional nodes and variability couplings were transformed into four directed weighted networks. The resulting in-degree/out-degree and in-weight/out-weight distributions are presented in Figure 6 and Figure 7, respectively.

The four networks were similar in overall scale, with average degrees ranging from 1.67 to 2. More than 71% of the nodes had in-degree and out-degree values below 3. The in-degree distributions were mainly concentrated between 1 and 2. Under *S*_1_, *F*_10_ exhibited the highest in-degree of 5, whereas the maximum in-degree decreased to 3 in the other three scenarios. Several terminal functions, including *F*_13_, *F*_14_, and *F*_15_, showed an in-degree of 1 and an out-degree of 0. The maximum out-degree increased from 6 in *S*_1_ to 8 in the outward-oriented scenarios.

The edge-weight distributions also differed across the four scenarios. *S*_1_ showed the highest maximum in-weight of 11 and a maximum out-weight of 10. The maximum in-weights decreased to 5 in *S*_2_–*S*_4_, while the maximum out-weights ranged from 7 to 9. These topology results provide the structural basis for the subsequent node-importance analysis.

#### 4.3.2. Structural Importance and Functional-Performance Distribution

The K-shell analysis produced distinct structural-importance patterns across the four surge scenarios, as summarized in Table 7. Under *S*_1_, *F*_20_ showed the highest structural importance (*ψ*_20_ = 39.56), followed by *F*_6_ and *F*_19_ (*ψ* = 37.67), and *F*_5_, *F*_10_ (*ψ* = 35.78). Under *S*_2_, *F*_3_ and *F*_7_ ranked first (*ψ* = 17.00), followed by *F*_4_ and *F*_17_ (*ψ* = 16.50). Under *S*_3_, *F*_5_ had the highest structural importance (*ψ* = 19.08), followed by *F*_4_ and *F*_19_ (*ψ* = 18.50), whereas under *S*_4,_ *F*_4_ ranked first (*ψ* = 18.77), followed by *F*_3_ and *F*_5_ (*ψ* = 18.23) and *F*_23_ (*ψ* = 17.69).

The functional-performance results also exhibited heterogeneity across nodes and scenarios. The mean performance values ranged from 0.46 to 0.78 in *S*_1_, from 0.52 to 0.76 in *S*_2_, from 0.50 to 0.80 in *S*_3_, and from 0.55 to 0.82 in *S*_4_. The corresponding standard deviations ranged from 0.14 to 0.22, 0.15 to 0.21, 0.14 to 0.22, and 0.13 to 0.21, respectively. The resulting root-node performance uncertainty was represented using truncated-normal distributions bounded within [0, 1], with the scenario specific parameters for the top 5 nodes ranked by structural importance reported in Table 7.

Structural importance and functional performance did not show a one-to-one correspondence. For example, *F*_20_ had the highest importance in *S*_1_ while maintaining a relatively high mean performance of 0.78, whereas *F*_10_ exhibited a substantially lower mean performance of 0.46. Similar differences were observed across the other scenarios, showing that structural importance and functional-performance uncertainty represent distinct characteristics of the modeled system.

Based on the structural contribution weights, the conditional performance functions of the intermediate capability nodes and the top-level surge-capacity node were subsequently specified, as presented in Table 8.

### 4.4. BN Inference Results

The BN models were implemented and evaluated using AgenaRisk. The inferred performance distributions of the five capability nodes and the overall hospital surge-capacity node *C* were obtained for the four scenarios. The value of *C* was used to represent the overall surge-capacity performance, with higher values indicating better modeled performance.

**Figure 8:**
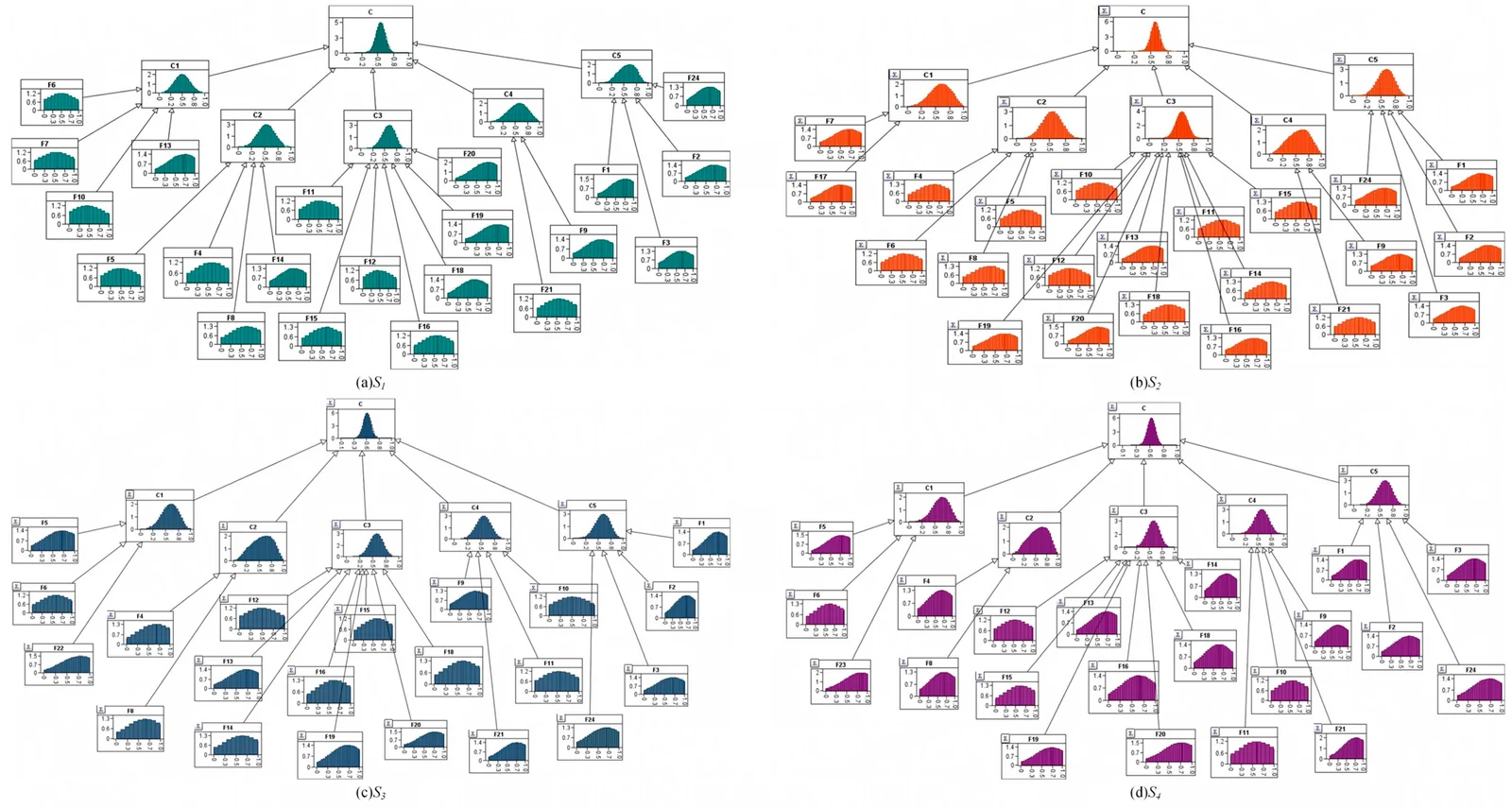
BN models and baseline surge-capacity performance under four scenarios.

The baseline BN inference results were shown in Figure 8. In terms of overall surge capacity *C*, the scenarios were ranked as *S*_4_(0.5867) > *S*_3_(0.5723) > *S*_2_(0.5631) > *S*_1_(0.5479). The difference between the highest and lowest overall performance was 0.0388. Differences were also observed across the five capability dimensions. Under *S*_1_, *C*_3_ showed the highest performance (0.5861), followed by *C*_5_(0.5778), *C*_4_(0.5624), *C*_2_(0.5334), and *C*_1_(0.5032). Under *S*_2_, *C*_1_ ranked highest (0.5943), followed by *C*_5_(0.5872), *C*_4_(0.5609), *C*_3_ (0.5539), and *C*_2_(0.5446). Under *S*_3_, the corresponding order was *C*_1_(0.5926), *C*_5_(0.5900), *C*_3_(0.5698), *C*_2_(0.5670), and *C*_4_(0.5525). Under *S*_4_, *C*_1_ again exhibited the highest performance (0.6193), followed by *C*_5_(0.5954), *C*_3_(0.5793), *C*_2_(0.5775), and *C*_4_(0.5699). The propagated output distributions also exhibited appreciable uncertainty. The standard deviations of overall *C* were 0.0733, 0.0638, 0.0670, and 0.0645 for *S*_1_–*S*_4_, respectively, while the corresponding uncertainty intervals were [0.4991–0.5977], [0.5208–0.6061], [0.5279–0.6179], and [0.5441–0.6305]. Similar uncertainty summaries were obtained for the five capability dimensions.

The uncertainty intervals of *C* overlapped across all scenario pairs. The pairwise overlap ranged from 0.408 to 0.805, with the greatest overlap between *S*_2_ and *S*_3_. Although the expected modeled performance followed the order *S*_4_ > *S*_3_ > *S*_2_ > *S*_1_, the scenario distributions were not sharply separated. The observed differences can therefore be interpreted as relative shifts in expected modeled performance rather than deterministic differences among the four surge configurations.

#### 4.4.1. BN Propagation Analysis

Forward propagation analysis was conducted using the BN model to evaluate the extent to which individual functional nodes affect surge capacity under different scenarios. Based on the baseline situation, the five highest-ranked functions in each scenario were sequentially perturbed. For each selected function, its performance distribution was reduced by 50%, while the structural weights and the distributions of the remaining functions were kept unchanged. The resulting changes in the five capability dimensions and overall surge-capacity performance are presented in Table 9.

As shown in Table 9, cumulative perturbations produced a progressive decline in overall surge-capacity performance across all four scenarios. From the baseline to situation 5, *C* decreased from 0.5479 to 0.4189 in *S*_1_, from 0.5631 to 0.4679 in *S*_2,_ from 0.5723 to 0.4549 in *S*_3_, and from 0.5867 to 0.4708 in *S*_4_, corresponding to reductions of 23.54%, 16.91%, 20.51%, and 19.75%, respectively. The capability-level results further show that the degradation pathways differed across scenarios: the decline in *S*_1_ was mainly transmitted through *C*_3_ and *C*_1_, *S*_2_ primarily through *C*_1_ and *C*_2_, *S*_3_ through *C*_1_, *C*_2_, and *C*_3_, and *S*_4_ through *C*_1_, *C*_2_, and *C*_5_. Overall, the results indicate that functional-performance degradation was progressively propagated from the root-function layer to the capability layer and ultimately to overall surge capacity, with the magnitude and pathway of degradation varying across the four modeled scenarios.

#### 4.4.2. Sensitivity Analysis

**Figure 9:**
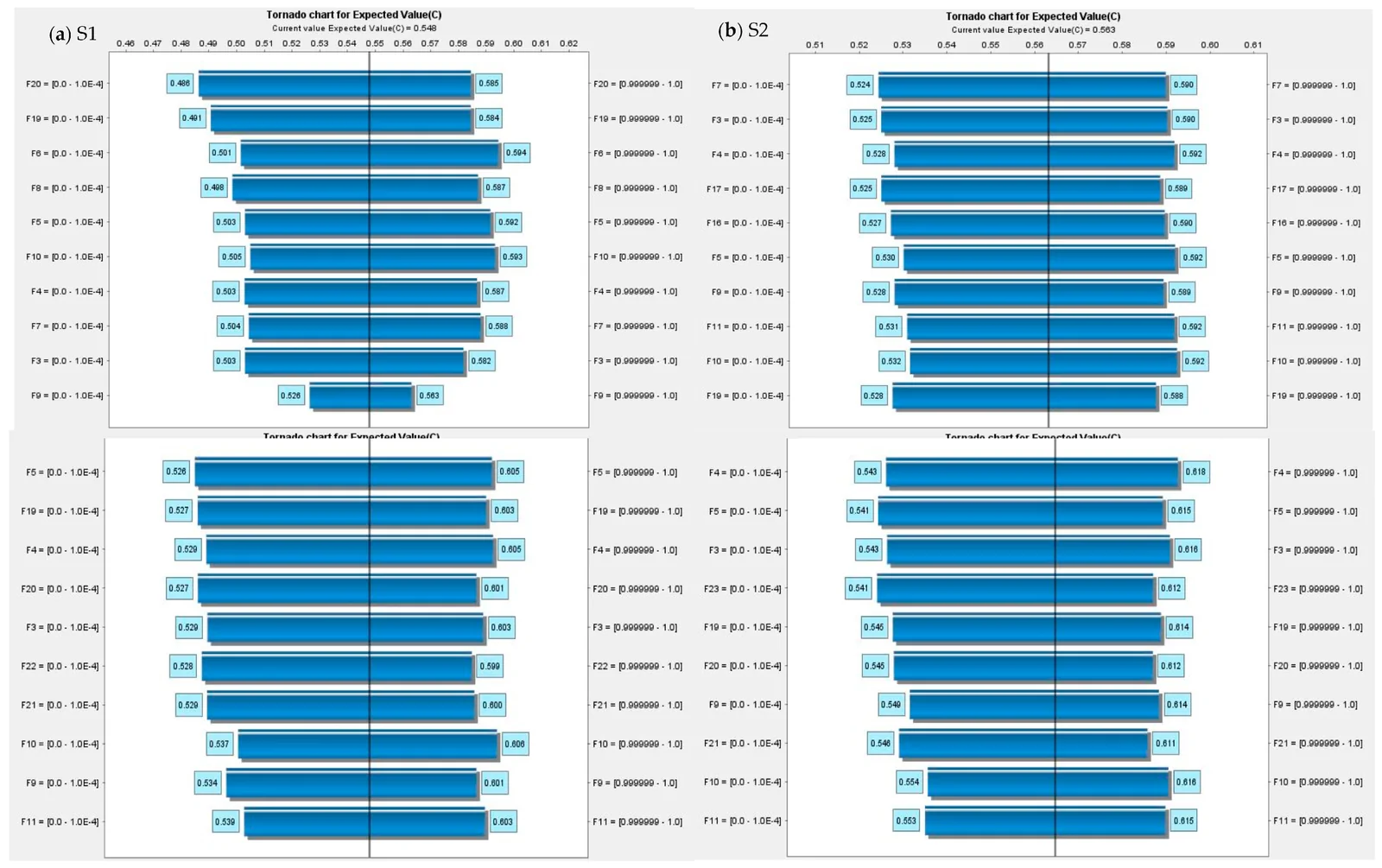
Root-node sensitivity analysis of hospital surge capability in different scenarios.

Sensitivity analysis was conducted with the overall surge-capacity performance *C* specified as the target node. As shown in Figure 9, the root-node sensitivity profiles varied across the four scenarios. In *S*_1_, *F*_20_ exhibited the greatest influence on *C*, followed by *F*_19_ and *F*_6_; in *S*_2_, the highest sensitivities were associated with *F*_7_, *F*_3_, and *F*_4_; in *S*_3_, *F*_5_, *F*_19_, and *F*_4_ ranked highest; and in *S*_4_, *F*_4_, *F*_5_, and *F*_3_ showed the greatest sensitivity. Overall, functions with relatively high structural importance generally also showed comparatively large propagated effects on *C* although the rankings were not identical, reflecting the distinction between structural importance and performance sensitivity.

**Figure 10:**
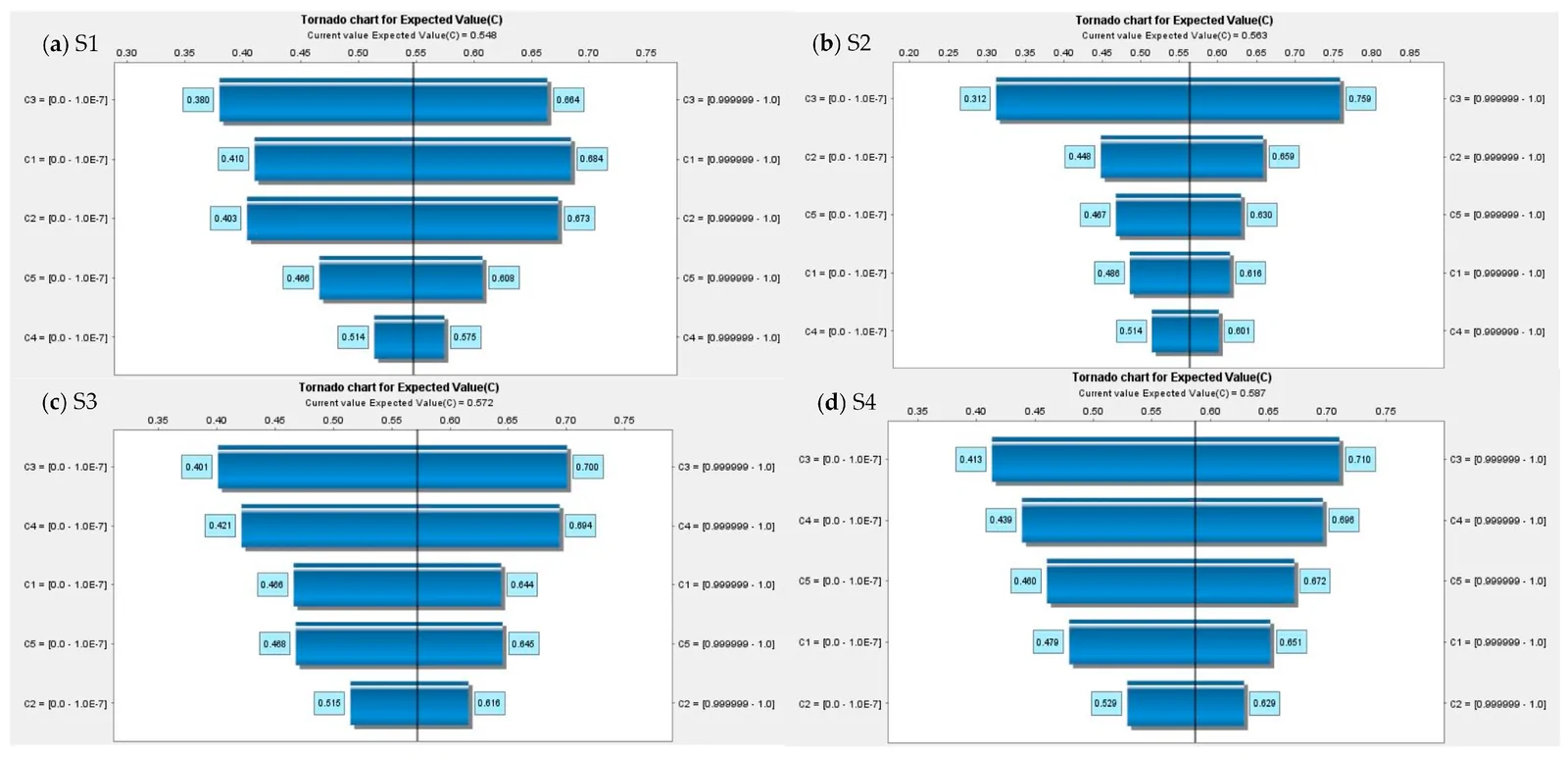
Sensitivity analysis of intermediate node of hospital surge capability in different scenarios.

The sensitivity analysis of the five capability nodes further revealed clear differences in their influence on overall surge-capacity performance (Figure 10). *C*_3_ exhibited the highest sensitivity in all four scenarios. This result can be interpreted within the specified model, the observed sensitivity reflects the combined effects of the predefined function–capability mapping and aggregation weights, together with the uncertainty propagated from the constituent functional-performance distributions. The secondary sensitivity patterns remained scenario dependent, with *S*_1_ showing greater influence from *C*_1_ and *C*_2_, *S*_2_ from *C*_2_, and *S*_3_ and *S*_4_ from *C*_4_. Overall, the results identified scenario-specific functional sensitivity patterns.

### 4.5. Robustness and Convergence Analysis

Robustness analyses were conducted by varying the Monte Carlo sample size, coupling criteria, root-node distributional assumptions, expert-derived parameters, and structural weighting parameters.

(1) Monte Carlo convergence.

The convergence of the Monte Carlo simulation was examined using 500, 1000, 2000, and 5000 iterations. The estimated values of *C* gradually stabilized as the number of iterations increased. At 500 iterations, *C* ranged from 0.5446 to 0.5824 across the four scenarios, whereas the corresponding values at 1000 iterations were 0.5479, 0.5631, 0.5723, and 0.5867 for *S*_1_–*S*_4_, respectively. Increasing the sample size to 5000 produced only minor changes, yielding 0.5491, 0.5640, 0.5732, and 0.5876. Relative to the 5000-iteration results, the deviations at 1000 iterations were 0.22%, 0.16%, 0.16%, and 0.15% for *S*_1_–*S*_4_, respectively, indicating satisfactory convergence.

(2) Coupling-identification assumptions.

The VPN threshold and exceedance criterion were varied to examine the robustness of the FRAM-derived network structure. As summarized in Table 10, changing the VPN threshold from the baseline value of 6 to 9 or 12 resulted in maximum changes in *C* of less than 1.0%, while varying the exceedance criterion from 5% to 2.5% or 10% produced similarly small changes. The scenario ordering *S*_4_ > *S*_3_ > *S*_2_ > *S*_1_ remained unchanged under all tested settings. Moreover, 9–11 of the 12 baseline top-three structurally important functions across the four scenarios remained within the corresponding top-three sets after the coupling criteria were altered.

(3) Root-node performance assumptions.

Alternative assumptions for the root-node performance distributions were also examined. First, direct propagation of the empirical distributions produced the same scenario ordering as the baseline truncated-normal model, indicating that the principal comparative result was not driven by the parametric approximation. Then, replacing the baseline with Beta distributions yielded *C* values of 0.5416, 0.5587, 0.5689, and 0.5825 for *S*_1_–*S*_4_, respectively, corresponding to deviations of less than 1.2% from the baseline results. In addition, the expert-derived mean performance parameters were randomly varied within ±10% over 100 repeated parameter sets. The mean resulting values of *C* were 0.5483, 0.5635, 0.5727, and 0.5870, and the baseline ordering was retained in 96 of the 100 parameter sets. The principal sensitive functions also showed substantial overlap with the baseline sensitivity results.

(4) Structural weighting assumptions.

The robustness of the K-shell aggregation weights was first assessed by independently varying the weights within ±10%, followed by renormalization. Across 100 repeated weight perturbations, the mean values of *C* remained close to the baseline estimates, and the scenario ordering was unchanged in all runs. The Spearman correlations between the baseline and perturbed functional sensitivity rankings ranged from 0.88 to 0.92 across the four scenarios, indicating relatively stable identification of the principal influential functions. To further examine whether the scenario level conclusions depended on the K-shell capability weights, an equal-weight benchmark was introduced at the capability aggregation level. Under this alternative specification, the *C* values were 0.5526, 0.5682, 0.5744, and 0.5883 for *S*_1_–*S*_4_, respectively. The maximum deviation from the baseline results was 0.90%, and the scenario ordering remained unchanged. These results indicate that the principal comparative findings were not sensitive to the specific capability level weighting rule.

## 5. Discussion

### 5.1. From Resource Expansion to Function–Capability Generation

The results support a view of hospital surge capacity as a system capability generated through the coordinated operation of multiple functions rather than as a direct consequence of increasing resource quantities. Conventional surge capacity research has largely emphasized beds, personnel, equipment, supplies, and their allocation, whereas such indicators provide limited information on whether mobilized resources can be effectively integrated into emergency operations and sustained under changing conditions. The function–capability framework developed in this study addresses this gap by explicitly connecting operational functions with five resilience-oriented capability dimensions and, ultimately, overall surge-capacity performance.

The findings further demonstrate the importance of distinguishing structural importance from functional performance. A function that occupies a structurally important position in the FRAM derived network does not necessarily exhibit high operational performance, and, conversely, a well-performing function may make only a limited structural contribution to the overall system. Overall surge capacity therefore emerges from the interaction between where a function is positioned in the system and how well that function performs. This distinction extends resource oriented surge assessment toward a more explicit representation of the mechanism through which available resources and organizational functions are transformed into system-level capabilities. This interpretation is consistent with the theoretical premise that surge capacity resources provide the basis for response, while resilience reflects how these resources are reorganized, coordinated, and sustained over time.

Compared with conventional surge-capacity assessments that primarily aggregate resource availability or preparedness indicators, the present framework explicitly represents how operational functions contribute to capability generation. It also differs from previous FRAM-BN applications that mainly use probabilistic reasoning to quantify functional variability or risk propagation, because structural importance and functional-performance uncertainty are treated as separate analytical dimensions before being integrated at the capability level. Likewise, compared with resilience assessment approaches based primarily on composite indicators, the proposed framework provides an explicit functional pathway linking operational activities to system-level performance. These differences constitute the principal methodological contribution of the present study.

### 5.2. Scenario-Specific Surge-Capacity Performance and Capability Patterns

The BN results showed moderate but consistent differences in modeled surge-capacity performance across the four configurations, with *C* increasing from 0.5479 in *S*_1_ to 0.5631 in *S*_2_, 0.5723 in *S*_3_, and 0.5867 in *S*_4_. These values should be interpreted as model-based estimates under the specified scenario structures and parameter assumptions, rather than empirical evidence that one surge strategy is universally superior to another. The four scenarios are synthesized organizational configurations rather than experimentally evaluated hospitals, which requires caution when generalizing their relative performance. This distinction directly addresses the need to separate model-based inference from empirically observed hospital performance.

Within the modeled configurations, however, the gradual increase from *S*_1_ to *S*_4_ suggests that broader functional integration and coordination can be associated with higher overall surge-capacity performance when the corresponding functional nodes maintain adequate operating performance. *S*_1_ relies primarily on rapid internal modular reconfiguration and showed comparatively lower *C*_1_ and *C*_2_, despite relatively high operational continuity *C*_3_. By contrast, S4 exhibited the highest modeled values for *C*_1_, *C*_2_, *C*_4_, and *C*_5_, producing the highest overall *C*. *S*_2_ and *S*_3_ occupied intermediate positions, but their capability profiles differed, indicating that similar overall performance may arise through different combinations of functional reconfiguration, resource configuration, continuity, regional coordination, and collaborative optimization.

This finding reinforces the value of assessing hospital surge capacity as a multidimensional capability profile rather than relying exclusively on a single composite score. The five capability dimensions were designed to bridge surge-capacity components and resilience characteristics, including adaptability, resourcefulness, robustness, continuity, and learning and transformation. Consequently, differences among surge configurations reflect not only variation in the amount of available resources but also differences in the pathways through which those resources are organized and converted into sustained emergency operations.

### 5.3. Structural Importance, Performance Sensitivity, and Management Implications

The sensitivity results provide additional insight into the functional pathways associated with overall surge-capacity performance. At the root-node level, the most influential functions varied substantially among scenarios: *F*_20_, *F*_19_, and *F*_6_ showed the largest propagated influence in *S*_1_; *F*_7_, *F*_3_, and *F*_4_ in *S*_2_; *F*_5_, *F*_19_, and *F*_4_ in *S*_3_; and *F*_4_, *F*_5_, and *F*_3_ in *S*_4_. This variation indicates that the functions most strongly associated with changes in overall surge performance depend on the organizational configuration of the surge system. Accordingly, the model does not support a single universal set of critical functions for all surge arrangements.

At the capability level, *C*_3_ consistently showed the strongest propagated sensitivity to *C* across the four scenarios. This pattern can be interpreted within the predefined function–capability and weighting architecture. Capability level sensitivity is jointly determined by the structural aggregation scheme and the stochastic uncertainty inherited from constituent functions. Therefore, the result indicates stronger modeled propagation through operational continuity under the specified architecture. The scenario-specific differences observed at the functional level further indicate that stochastic performance uncertainty interacts with the structural configuration to produce different sensitivity profiles. These differences suggest that operational continuity represents a common high sensitivity pathway within the modeled configurations, whereas the additional capabilities contributing to system performance depend on how the surge response is organized.

The propagation analysis further showed that cumulative degradation of key functional nodes reduced *C* by 23.54% in *S*_1_, 16.91% in *S*_2_, 20.51% in *S*_3_, and 19.75% in *S*_4_. Rather than demonstrating causal effects, these results illustrate how functional-performance deterioration can be transmitted through the modeled capability structure. From a management perspective, the findings suggest that preparedness should combine protection of configuration-specific critical functions with maintenance of cross-cutting operational continuity. Thus, surge planning should not focus only on expanding beds, personnel, or equipment; it should also consider whether supporting functions, information flows, clinical continuity, coordination mechanisms, and organizational interfaces can maintain adequate performance as demand increases.

### 5.4. Limitations

Several limitations should be considered when interpreting the findings. First, the four surge scenarios are synthesized configurations derived from recurring organizational practices and expert-informed descriptions rather than prospective observations of four individual hospitals. The resulting differences should therefore be interpreted as scenario-based model outputs rather than direct evidence of the relative effectiveness of real-world surge strategies. Second, although the model development was informed not only by expert elicitation but also by hospital operational data, emergency response reports, and related documentary evidence, these empirical sources were primarily used to support scenario construction, functional identification, and parameter specification rather than as an independent validation dataset. The robustness analyses do not constitute external predictive or criterion validation. Therefore, the transferability of the framework across institutional contexts remain to be established. Validation against independently observed hospital performance or external hospital datasets should be regarded as a necessary next step. Third, the relationships represented in the FRAM and BN encode functional dependencies identified from theory, expert judgment, and scenario modeling; they should not be interpreted as independently established causal relationships. Fourth, the current study evaluates surge-capacity performance within bounded scenario configurations and does not directly model longer-term organizational transformation, recovery trajectories, or broader sustainability outcomes. Future studies could incorporate longitudinal operational data, dynamic BN structures, real-time hospital information systems, and multi-hospital validation to examine how surge capabilities evolve over time and across different institutional contexts.

## 6. Conclusions

This study developed a resilience-oriented function–capability framework for assessing hospital surge capacity by integrating hospital surge capacity theory, resilience theory, the FRAM, complex network analysis, and BN inference. The framework establishes a hierarchical relationship among surge components, operational functions, five capability dimensions, and surge-capacity performance. The main conclusions are as follows.

(1) Hospital surge capacity should be understood as a systemic capability generated through the coordinated operation of multiple functions rather than as a direct consequence of resource expansion. By explicitly linking resource and organizational components to operational functions and capability outcomes, the proposed framework extends conventional resource oriented assessment toward a function–capability perspective.
(2) Five capability dimensions—functional reconfiguration, resource configuration, operational continuity, regional coordination, and collaborative optimization—provide a multidimensional representation of hospital surge performance. The revised modeling results further distinguish structural importance from functional performance: K-shell importance characterizes the structural contribution of individual functions, whereas the BN represents uncertainty in their operating performance. Their integration enables functional conditions to be propagated to capability level and overall surge-capacity performance.
(3) The four modeled surge configurations exhibited different capability profiles and overall performance levels. The estimated overall surge-capacity values were 0.5479, 0.5631, 0.5723, and 0.5867 for *S*_1_–*S*_4_, respectively. At the functional level, the most influential nodes varied across configurations, whereas *C*_3_ consistently exhibited the greatest sensitivity to overall surge-capacity performance. The cumulative perturbation analysis further showed that degradation of structurally important functions progressively reduced system performance through different capability pathways.
(4) The integration of FRAM, improved K-shell analysis, and BN provides a structured approach for connecting functional interactions, structural importance, performance uncertainty, and system-level capability assessment. Robustness analyses showed that the main scenario ordering and identification of principal influential nodes remained generally stable. The results therefore provide a model-based basis for identifying configuration-specific functional vulnerabilities and capability priorities.

## Figures and Tables

**Table 1:** Experts in relevant fields and their role characteristics.

Participating Experts	Experience (Years)	Number	Role	Institution Type
Hospital managers	15–20	8	Determine the functions, six dimensions and working conditions; identify function variability and analyze risk scenarios; determine key coupling	Three tertiary general hospitals
Emergency rescuer	10–20	9		Municipal medical emergency response team/Hospital
Treatment physicians	10–20	4		Three tertiary general hospitals
Public health emergency managers	20–30	4		Emergency Medical Rescue Center/Hospital

**Table 2:** Summary of Interview Outline.

Interview Number	Question
First round	What are the key functions included in the hospital surge progress? What are the main purposes and boundaries of each function? What are the Input, Output, Preconditions, Resources, Time and Control for each function? Is there any functional omission, duplication, or unclear definition?
Second round	What are the typical scenarios for sudden medical demand in hospitals? What functions have changed in different scenarios? What are the coupling relationships between different functions and which functions are prone to variability? What are the main manifestations of functional variation in different scenarios?

**Table 3:** Four scenarios for hospital surge capacity.

Code	Scenario	Description	Characteristics
S_1_	Modularization surge	A way of surge where hospitals restructure resources across departments by rapidly adjusting internal medical functional modules based on existing in-house resources.	Internal resources, Modularization
S_2_	Unitization surge	A way of surge where hospitals construct separate operational units amid resource limits for closed-loop operation and improved continuous medical treatment capacity.	Internal resources, Unitization
S_3_	Specialization surge	A way of surge where hospitals dispatch specialized medical teams to target zones for specific missions to quickly strengthen required medical capabilities.	External resources, Modularization
S_4_	Integrated surge	A way of surge where integrated medical formations join emergency rescue per mission demands for rapid delivery of holistic medical capacity.	External resources, Unitization

**Table 4:** FRAM function description and coupling variability identification results.

Scenario	Function	Description	Variability Coupling
*S*_1_	*F*_1_	Risk perception	*F*_1_(O) *→ F*_2_(I)
	*F*_2_	Emergency response	*F*_1_(O) *→ F*_2_(O) *→ F*_3_(I)
	*F*_3_	Surge mode judgment/switch	*F*_2_(O) → *F*_3_(O) *→ F*_4_(I)*, F*_5_(I)*, F*_7_(I)*, F*_8_(I)*, F*_9_(P)*, F*_11_(P)
	*F*_4_	Emergency resource mobilization	*F*_3_(O) → *F*_4_(O) → *F*_5_(I), *F*_8_(I), *F*_14_(P)
	*F*_5_	Medical personnel organization	*F*_3_(O), *F*_4_(O) → *F*_5_(O) → *F*_6_(I), *F*_12_(P), *F*_19_(I)
	*F*_6_	Emergency training and ability adaptation	*F*_5_(O), *F*_20_(O) → *F*_6_(O) → *F*_10_(P), *F*_12_(I)
	*F*_7_	Space reconstruction and ward conversion	*F*_3_(O) → *F*_7_(O) → *F*_8_(P), *F*_10_(P), *F*_15_(P), *F*_17_(I)
	*F*_8_	Medical equipment configuration	*F*_3_(O), *F*_4_(O), *F*_7_(O) → *F*_8_(O) → *F*_10_(P), *F*_13_(P)
	*F*_9_	Triage and triage of patients	*F*_3_(O) → *F*_9_(O) → *F*_10_(I), *F*_11_(I)
	*F*_10_	Critical care treatment	*F*_6_(O), *F*_7_(O), *F*_8_(O), *F*_9_(O) → *F*_10_(O) → *F*_18_(I)
	*F*_11_	Routine treatment maintenance	*F*_9_(O), *F*_3_(O) → *F*_11_(O) → *F*_18_(I)
	*F*_12_	Nursing support	*F*_6_(O), *F*_10_(O), *F*_5_(P) → *F*_12_(O)
	*F*_13_	Medical technical support	*F*_8_(O) → *F*_13_(O) → *F*_10_(I), *F*_11_(I)
	*F*_14_	Drug and supply provision	*F*_4_(O) → *F*_14_(O) → *F*_10_(I), *F*_11_(I)
	*F*_15_	Logistics operations support	*F*_7_(O) → *F*_15_(O) → *F*_10_(R), *F*_11_(R)
	*F*_16_	Infection prevention and control	*F*_20_(O) → *F*_16_(O) → *F*_10_(C), *F*_11_(C), *F*_18_(P), *F*_19_(P)
	*F*_17_	In-hospital unit isolation	*F*_7_(O) → *F*_17_(O) → *F*_9_(P), *F*_10_(P), *F*_11_(P)
	*F*_18_	Medical waste disposal	*F*_10_(O), *F*_11_(O), *F*_16_(P) → *F*_18_(O) → *F*_16_(I)
	*F*_19_	Medical health monitoring	*F*_5_(O), *F*_16_(O), *F*_20_(O) → *F*_19_(O) → *F*_5_(C), *F*_24_(I)
	*F*_20_	Information collection and transmission	*F*_1_(O) *→ F*_20_(O) *→ F*_6_(P)*, F*_16_(P)*, F*_19_(P)*, F*_24_(I)
	*F*_21_	External collaborative docking	*F*_20_(O) *→ F*_21_(O) *→ F*_4_(I)*, F*_6_(P)
	*F*_24_	Surge feedback and adjustment	*F*_20_(O)*, F*_19_(O) *→ F*_24_(O) *→ F*_2_(I)*, F*_3_(C)
*S*_2_	*F*_1_	Risk perception	*F*_1_(O) *→ F*_2_(I)
	*F*_2_	Emergency response	*F*_1_(O) *→ F*_2_(O) *→ F*_3_(I)*, F*_24_(C)
	*F*_3_	Surge mode judgment/switch	*F*_2_(*O*) *→ F*_3_(*O*) *→ F*_4_(I), *F*_5_(I), *F*_7_(I), *F*_9_(P), *F*_17_(I), *F*_22_(I), *F*_23_(I)
	*F*_4_	Emergency resource mobilization	*F*_3_(O) → *F*_4_(O) → *F*_5_(I), *F*_8_(I), *F*_14_(P)
	*F*_5_	Medical personnel organization	*F*_3_(O), *F*_4_(O) → *F*_5_(O) → *F*_6_(I), *F*_12_(P), *F*_19_(I)
	*F*_6_	Emergency training and ability adaptation	*F*_5_(O), *F*_20_(O) → *F*_6_(O) → *F*_10_(P), *F*_11_(P)
	*F*_7_	Space reconstruction and ward conversion	*F*_3_(O) → *F*_7_(O) → *F*_17_(I), *F*_15_(P)
	*F*_8_	Medical equipment configuration	*F*_4_(O), *F*_7_(O) → *F*_8_(O) → *F*_10_(R), *F*_11_(R), *F*_13_(P)
	*F*_9_	Triage and triage of patients	*F*_17_(O) → *F*_9_(O) → *F*_10_(I), *F*_11_(I)
	*F*_10_	Critical care treatment	*F*_9_(O), *F*_17_(O), *F*_16_(O) → *F*_10_(O) → *F*_18_(I), *F*_12_(I)
	*F*_11_	Routine treatment maintenance	*F*_9_(O), *F*_17_(O), *F*_16_(O) → *F*_11_(O) → *F*_18_(I), *F*_12_(I)
	*F*_12_	Nursing support	*F*_10_(O), *F*_11_(O), *F*_5_(O) → *F*_12_(O)
	*F*_13_	Medical technical support	*F*_8_(O), *F*_17_(O) → *F*_13_(O) → *F*_10_(I), *F*_11_(I)
	*F*_14_	Drug and supply provision	*F*_4_(O), *F*_17_(O) → *F*_14_(O) → *F*_10_(I), *F*_11_(I)
	*F*_15_	Logistics operations support	*F*_7_(O), *F*_17_(O) → *F*_15_(O) → *F*_10_(R), *F*_11_(R), *F*_20_(R)
	*F*_16_	Infection prevention and control	*F*_17_(O), *F*_20_(O) → *F*_16_(O) → *F*_10_(C), *F*_11_(C), *F*_18_(P), *F*_19_(P)
	*F*_17_	In-hospital unit isolation	*F*_3_(O), *F*_7_(O) → *F*_17_(O) → *F*_8_(P), *F*_9_(P), *F*_10_(P), *F*_11_(P), *F*_13_(P), *F*_14_(P), *F*_15_(P), *F*_16_(I)
	*F*_18_	Medical waste disposal	*F*_10_(O), *F*_11_(O), *F*_17_(P) → *F*_18_(O) → *F*_16_(I)
	*F*_19_	Medical health monitoring	*F*_5_(O), *F*_16_(O) → *F*_19_(O) → *F*_24_(I)
	*F*_20_	Information collection and transmission	*F*_1_(O), *F*_20_(O) → *F*_6_(P), *F*_16_(P), *F*_15_(R)
	*F*_21_	External collaborative docking	*F*_20_(O) → *F*_21_(O) → *F*_4_(I)
	*F*_24_	Surge feedback and adjustment	*F*_20_(O), *F*_19_(O) → *F*_24_(O) → *F*_2_(I)
*S*_3_	*F*_1_	Risk perception	*F*_1_(O) → *F*_2_(I)
	*F*_2_	Emergency response	*F*_1_(O) → *F*_2_(O) → *F*_3_(I)
	*F*_3_	Surge mode judgment/switch	*F*_2_(O) → *F*_3_(O) → *F*_4_(I), *F*_5_(I), *F*_22_(I)
	*F*_4_	Emergency resource mobilization	*F*_3_(O), *F*_21_(O) → *F*_4_(O) → *F*_5_(I), *F*_8_(I), *F*_14_(P)
	*F*_5_	Medical personnel organization	*F*_3_(O), *F*_4_(O) → *F*_5_(O) → *F*_6_(I), *F*_12_(P), *F*_19_(I), *F*_22_(I)
	*F*_6_	Emergency training and ability adaptation	*F*_5_(O), *F*_21_(O) → *F*_6_(O) → *F*_9_(P), *F*_10_(P), *F*_11_(P)
	*F*_8_	Medical equipment configuration	*F*_4_(O) → *F*_8_(O) → *F*_13_(P), *F*_22_(R)
	*F*_9_	Triage and triage of patients	*F*_22_(O), *F*_21_(O) → *F*_9_(O) → *F*_10_(I), *F*_11_(I)
	*F*_10_	Critical care treatment	*F*_9_(O), *F*_6_(O), *F*_21_(O) → *F*_10_(O) → *F*_18_(I), *F*_24_(I)
	*F*_11_	Routine treatment maintenance	*F*_9_(O), *F*_22_(O) → *F*_11_(O) → *F*_18_(I)
	*F*_12_	Nursing support	*F*_10_(O), *F*_5_(O) → *F*_12_(O)
	*F*_13_	Medical technical support	*F*_8_(O) → *F*_13_(O) → *F*_10_(I), *F*_11_(I)
	*F*_14_	Drug and supply provision	*F*_4_(O), *F*_21_(O) → *F*_14_(O) → *F*_10_(I), *F*_11_(I)
	*F*_15_	Logistics operations support	*F*_21_(O) → *F*_15_(O) → *F*_10_(R), *F*_19_(R), *F*_20_(R)
	*F*_16_	Infection prevention and control	*F*_20_(O) → *F*_16_(O) → *F*_10_(C), *F*_11_(C), *F*_19_(P)
	*F*_18_	Medical waste disposal	*F*_10_, *F*_11_(O), *F*_21_(O) → *F*_18_(O)
	*F*_19_	Medical health monitoring	*F*_5_(O), *F*_16_(O), *F*_20_(O) → *F*_19_(O) → *F*_24_(I)
	*F*_20_	Information collection and transmission	*F*_1_(O) → *F*_20_(O) → *F*_16_(P), *F*_19_(P), *F*_24_(I)
	*F*_21_	External collaborative docking	*F*_20_(O) → *F*_21_(O) → *F*_4_(I), *F*_6_(P), *F*_9_(P), *F*_10_(P), *F*_14_(P), *F*_15_(P), *F*_18_(P), *F*_22_(P)
	*F*_22_	Special medical team dispatched	*F*_3_(O), *F*_5_(O), *F*_8_(O) → *F*_22_(O) → *F*_9_(P), *F*_10_(P), *F*_11_(P)
	*F*_24_	Surge mode judgment/switch	*F*_20_(O), *F*_10_(O), *F*_19_(O) → *F*_24_(O) → *F*_2_(I), *F*_3_(C)
*S*_4_	*F*_1_	Risk perception	*F*_1_(O) → *F*_2_(I)
	*F*_2_	Emergency response	*F*_1_(O) → *F*_2_(O) → *F*_3_(I)
	*F*_3_	Surge mode judgment/switch	*F*_2_(O) → *F*_3_(O) → *F*_4_(I), *F*_5_(I), *F*_23_(I)
	*F*_4_	Emergency resource mobilization	*F*_3_(O), *F*_21_(O) → *F*_4_(O) → *F*_8_(I), *F*_14_(P), *F*_23_(R)
	*F*_5_	Medical personnel organization	*F*_3_(O), *F*_2_(C) → *F*_5_(O) → *F*_6_(I), *F*_12_(P), *F*_19_(I), *F*_23_(I)
	*F*_6_	Emergency training and ability adaptation	*F*_5_(O), *F*_23_(O) → *F*_6_(O) → *F*_10_(P), *F*_11_(P)
	*F*_8_	Medical equipment configuration	*F*_4_(O) → *F*_8_(O) → *F*_13_(P), *F*_23_(R)
	*F*_9_	Triage and triage of patients	*F*_23_(O), *F*_21_(O) → *F*_9_(O) → *F*_10_(I), *F*_11_(I)
	*F*_10_	Critical care treatment	*F*_9_(O), *F*_23_(O), *F*_21_(O) → *F*_10_(O) → *F*_18_(I), *F*_12_(I)
	*F*_11_	Routine treatment maintenance	*F*_9_(O), *F*_23_(O) → *F*_11_(O) → *F*_18_(I), *F*_12_(I)
	*F*_12_	Nursing support	*F*_5_(O), *F*_10_(O), *F*_11_(O) → *F*_12_(O)
	*F*_13_	Medical technical support	*F*_8_(O), *F*_23_(O) → *F*_13_(O) → *F*_10_(I), *F*_11_(I)
	*F*_14_	Drug and supply provision	*F*_4_(O), *F*_21_(O) → *F*_14_(O) → *F*_10_(I), *F*_11_(I)
	*F*_15_	Logistics operations support	*F*_21_(O), *F*_23_(O) → *F*_15_(O) → *F*_10_(R), *F*_13_(R), *F*_20_(R)
	*F*_16_	Infection prevention and control	*F*_23_(O), *F*_20_(O) → *F*_16_(O) → *F*_10_(C), *F*_11_(C), *F*_19_(P)
	*F*_18_	Medical waste disposal	*F*_10_(O), *F*_11_(O), *F*_21_(P) → *F*_18_(O) → *F*_16_(I)
	*F*_19_	Medical health monitoring	*F*_5_(O), *F*_23_(O), *F*_16_(P) → *F*_19_(O) → *F*_24_(I)
	*F*_20_	Information collection and transmission	*F*_1_(O) → *F*_20_(O) → *F*_16_(P), *F*_19_(P), *F*_24_(I)
	*F*_21_	External collaborative docking	*F*_20_(O) → *F*_21_(O) → *F*_4_(I), *F*_6_(P), *F*_9_(P), *F*_10_(P), *F*_14_(P), *F*_15_(P), *F*_18_(P), *F*_23_(P)
	*F*_23_	Integrated formation rescue team dispatched	*F*_3_(O), *F*_5_(O), *F*_8_(O) → *F*_23_(O) → *F*_9_(P), *F*_10_(P), *F*_11_(P)
	*F*_24_	Surge mode judgment/switch	*F*_20_(O), *F*_10_(O), *F*_19_(O) → *F*_24_(O) → *F*_2_(I), *F*_3_(C)

**Table 5:** Quantitative scoring criteria of each parameter in variability factors.

$V_{\mathrm{aj}}^{T}$		$V_{\mathrm{aj}}^{P}$		$V_{\mathrm{aj}}^{I}$		$CV_{\mathrm{ija}}^{T/P/I}$		*W_s_*		*V_ija_*	
On time	1	Precise	1	Normal	1	Dampening	0.5	No effect	1	No effect	1
Early	2	Acceptable	2	Strong	2	No effect	1	Low effect	2	Low effect	2
Late	3	Imprecise	3	Weak	3	Amplifying	2	High effect	4	High effect	4
No effect	4	Wrong	4								

**Table 6:** Functional characterization of hospital surge capacity under different scenarios.

Scenario	Hospital Surge Capacity	Functions Characterization
*S*_1_	$C_{1}$	*g*_1_(*F*_6_, *F*_7_, *F*_10_, *F*_13_)
	$C_{2}$	*g*_2_(*F*_4_, *F*_5_, *F*_8_, *F*_14_)
	$C_{3}$	*g*_3_(*F*_11_, *F*_12_, *F*_15_, *F*_16_, *F*_18_, *F*_19_, *F*_20_)
	$C_{4}$	*g*_4_(*F*_9_, *F*_21_)
	$C_{5}$	*g*_5_(*F*_1_, *F*_2_, *F*_3_, *F*_24_)
*S*_2_	$C_{1}$	*g*_1_(*F*_7_, *F*_17_)
	$C_{2}$	*g*_2_(*F*_4_, *F*_5_, *F*_6_, *F*_8_)
	$C_{3}$	*g*_3_(*F*_10_, *F*_11_, *F*_12_, *F*_13_, *F*_14_, *F*_15_, *F*_16_, *F*_18_, *F*_19_, *F*_20_)
	$C_{4}$	*g*_4_(*F*_9_, *F*_21_)
	$C_{5}$	*g*_5_(*F*_1_, *F*_2_, *F*_3_, *F*_24_)
*S*_3_	$C_{1}$	*g*_1_(*F*_5_, *F*_6_, *F*_22_)
	$C_{2}$	*g*_2_(*F*_4_, *F*_8_)
	$C_{3}$	*g*_3_(*F*_12_, *F*_13_, *F*_14_, *F*_15_, *F*_16_, *F*_18_, *F*_19_, *F*_20_)
	$C_{4}$	*g*_4_(*F*_9_, *F*_10_, *F*_11_, *F*_21_)
	$C_{5}$	*g*_5_(*F*_1_, *F*_2_, *F*_3_, *F*_24_)
*S*_4_	$C_{1}$	*g*_1_(*F*_5_, *F*_6_, *F*_23_)
	$C_{2}$	*g*_2_(*F*_4_, *F*_8_)
	$C_{3}$	*g*_3_(*F*_12_, *F*_13_, *F*_14_, *F*_15_, *F*_16_, *F*_18_, *F*_19_, *F*_20_)
	$C_{4}$	*g*_4_(*F*_9_, *F*_10_, *F*_11_, *F*_21_)
	$C_{5}$	*g*_5_(*F*_1_, *F*_2_, *F*_3_, *F*_24_)

**Table 7:** Structural importance and truncated-normal functional-performance distributions under different surge scenarios.

Scenario	Function	Importance *ψ_i_*	Rank	Mean *μ_i_*	SD *s_i_*	Truncated-Normal Distribution
*S*_1_	*F*_20_	39.5556	1	0.7800	0.1401	TN(0.7800, 0.1401; 0, 1)
	*F*_19_	37.6667	2	0.7500	0.1499	TN(0.7500, 0.1499; 0, 1)
	*F*_6_	37.6667	3	0.5001	0.2100	TN(0.5001, 0.2100; 0, 1)
	*F*_10_	35.7778	4	0.4603	0.2198	TN(0.4603, 0.2198; 0, 1)
	*F*_5_	35.7778	5	0.5204	0.2098	TN(0.5204, 0.2098; 0, 1)
*S*_2_	*F*_3_	17.0000	1	0.7000	0.1598	TN(0.7000, 0.1598; 0, 1)
	*F*_7_	17.0000	2	0.7201	0.1600	TN(0.7201, 0.1600; 0, 1)
	*F*_4_	16.5000	3	0.6300	0.1798	TN(0.6300, 0.1798; 0, 1)
	*F*_17_	16.5000	4	0.7397	0.1602	TN(0.7397, 0.1602; 0, 1)
	*F*_5_	16.0000	5	0.5902	0.1901	TN(0.5902, 0.1901; 0, 1)
*S*_3_	*F*_5_	19.0833	1	0.7201	0.1700	TN(0.7201, 0.1700; 0, 1)
	*F*_19_	18.5000	2	0.7302	0.1599	TN(0.7302, 0.1599; 0, 1)
	*F*_4_	18.5000	3	0.6802	0.1801	TN(0.6802, 0.1801; 0, 1)
	*F*_20_	17.9167	4	0.7698	0.1499	TN(0.7698, 0.1499; 0, 1)
	*F*_3_	17.9167	5	0.7102	0.1598	TN(0.7102, 0.1598; 0, 1)
*S*_4_	*F*_4_	18.7692	1	0.7001	0.1701	TN(0.7001, 0.1701; 0, 1)
	*F*_3_	18.2308	2	0.7202	0.1599	TN(0.7202, 0.1599; 0, 1)
	*F*_5_	18.2308	3	0.7801	0.1501	TN(0.7801, 0.1501; 0, 1)
	*F*_23_	17.6923	4	0.8210	0.1344	TN(0.8210, 0.1344; 0, 1)
	*F*_19_	17.1538	5	0.7502	0.1601	TN(0.7502, 0.1601; 0, 1)

**Table 8:** Conditional performance functions of intermediate and top nodes under different scenarios.

Scenario	Node	Conditional Performance Function
*S*_1_	*C*_1_	0.3477 × *F*_6_ + 0.3128 × *F*_7_ + 0.3303 × *F*_10_ + 0.0092 × *F*_13_
	*C*_2_	0.3184 × *F*_4_ + 0.3361 × *F*_5_ + 0.3361 × *F*_8_ + 0.0094 × *F*_14_
	*C*_3_	0.0592 × *F*_11_ + 0.1333 × *F*_12_ + 0.0089 × *F*_15_ + 0.0592 × *F*_16_ + 0.0533 × *F*_18_ + 0.3346 × *F*_19_ + 0.3515 × *F*_20_
	*C*_4_	0.6061 × *F*_9_ + 0.3939 × *F*_21_
	*C*_5_	0.1385 × *F*_1_ + 0.1688 × *F*_2_ + 0.5541 × *F*_3_ + 0.1386 × *F*_24_
	*C*	0.2643 × *C*_1_ + 0.2597 × *C*_2_ + 0.2746 × *C*_3_ + 0.0604 × *C*_4_ + 0.1410 × *C*_5_
*S*_2_	*C*_1_	0.5075 × *F*_7_ + 0.4925 × *F*_17_
	*C*_2_	0.3084 × *F*_4_ + 0.2991 × *F*_5_ + 0.1121 × *F*_6_ + 0.2804 × *F*_8_
	*C*_3_	0.1403 × *F*_10_ + 0.1403 × *F*_11_ + 0.1357 × *F*_12_ + 0.0090 × *F*_13_ + 0.0090 × *F*_14_ + 0.0090 × *F*_15_ + 0.1448 × *F*_16_ + 0.1357 × *F*_18_ + 0.1403 × *F*_19_ + 0.1359 × *F*_20_
	*C*_4_	0.7059 × *F*_9_ + 0.2941 × *F*_21_
	*C*_5_	0.1882 × *F*_1_ + 0.2706 × *F*_2_ + 0.4000 × *F*_3_ + 0.1412 × *F*_24_
	*C*	0.1275 × *C*_1_ + 0.2037 × *C*_2_ + 0.4207 × *C*_3_ + 0.0863 × *C*_4_ + 0.1618 × *C*_5_
*S*_3_	*C*_1_	0.4499 × *F*_5_ + 0.1415 × *F*_6_ + 0.4086 × *F*_22_
	*C*_2_	0.7551 × *F*_4_ + 0.2449 × *F*_8_
	*C*_3_	0.2130 × *F*_12_ + 0.0142 × *F*_13_ + 0.0142 × *F*_14_ + 0.0142 × *F*_15_ + 0.0142 × *F*_16_ + 0.2130 × *F*_18_ + 0.2627 × *F*_19_ + 0.2545 × *F*_20_
	*C*_4_	0.2456 × *F*_9_ + 0.2544 × *F*_10_ + 0.2367 × *F*_11_ + 0.2633 × *F*_21_
	*C*_5_	0.1843 × *F*_1_ + 0.2649 × *F*_2_ + 0.4127 × *F*_3_ + 0.1381 × *F*_24_
	*C*	0.1720 × *C*_1_ + 0.0994 × *C*_2_ + 0.2856 × *C*_3_ + 0.2670 × *C*_4_ + 0.1760 × *C*_5_
*S*_4_	*C*_1_	0.4349 × *F*_5_ + 0.1431 × *F*_6_ + 0.4220 × *F*_23_
	*C*_2_	0.7578 × *F*_4_ + 0.2422 × *F*_8_
	*C*_3_	0.2120 × *F*_12_ + 0.0141 × *F*_13_ + 0.0565 × *F*_14_ + 0.0141 × *F*_15_ + 0.0141 × *F*_16_ + 0.2120 × *F*_18_ + 0.2424 × *F*_19_ + 0.2348 × *F*_20_
	*C*_4_	0.2543 × *F*_9_ + 0.2457 × *F*_10_ + 0.2457 × *F*_11_ + 0.2543 × *F*_21_
	*C*_5_	0.1517 × *F*_1_ + 0.2181 × *F*_2_ + 0.3457 × *F*_3_ + 0.2845 × *F*_24_
	*C*	0.1654 × *C*_1_ + 0.0977 × *C*_2_ + 0.2793 × *C*_3_ + 0.2495 × *C*_4_ + 0.2081 × *C*_5_

**Table 9:** BN propagation results under cumulative perturbations of functional performance.

Scenario	Simulation	Node Performance (↓ 50%)					*C*_1_	*C*_2_	*C*_3_	*C*_4_	*C*_5_	*C*
		*F*_20_	*F*_19_	*F*_6_	*F*_10_	*F*_5_						
*S*_1_	Base	—	—	—	—	—	0.5032	0.5334	0.5861	0.5624	0.5778	0.5479
	s1	↓	—	—	—	—	0.5032	0.5334	0.4670 (↓)	0.5624	0.5778	0.5152 (↓)
	s2	↓	↓	—	—	—	0.5032	0.5334	0.3576 (↓)	0.5624	0.5778	0.4851 (↓)
	s3	↓	↓	↓	—	—	0.4163 (↓)	0.5334	0.3576	0.5624	0.5778	0.4621 (↓)
	s4	↓	↓	↓	↓	—	0.3368 (↓)	0.5334	0.3576	0.5624	0.5778	0.4411 (↓)
	s5	↓	↓	↓	↓	↓	0.3368	0.4478 (↓)	0.3576	0.5624	0.5778	0.4189 (↓)
*S*_2_		*F*_3_	*F*_7_	*F*_4_	*F*_17_	*F*_5_						
	base	—	—	—	—	—	0.5943	0.5446	0.5539	0.5609	0.5872	0.5631
	s1	↓	—	—	—	—	0.5943	0.5446	0.5539	0.5609	0.4634 (↓)	0.5431 (↓)
	s2	↓	↓	—	—	—	0.4344 (↓)	0.5446	0.5539	0.5609	0.4634	0.5227 (↓)
	s3	↓	↓	↓	—	—	0.4344	0.4565 (↓)	0.5539	0.5609	0.4634	0.5048 (↓)
	s4	↓	↓	↓	↓	—	0.2764 (↓)	0.4565	0.5539	0.5609	0.4634	0.4846 (↓)
	s5	↓	↓	↓	↓	↓	0.2764	0.3746 (↓)	0.5539	0.5609	0.4634	0.4679 (↓)
*S*_3_		*F*_5_	*F*_19_	*F*_4_	*F*_20_	*F*_3_						
	base	—	—	—	—	—	0.5926	0.5670	0.5698	0.5525	0.5900	0.5723
	s1	↓	—	—	—	—	0.4523 (↓)	0.5670	0.5698	0.5525	0.5900	0.5482 (↓)
	s2	↓	↓	—	—	—	0.4523	0.5670	0.4861 (↓)	0.5525	0.5900	0.5243 (↓)
	s3	↓	↓	↓	—	—	0.4523	0.3410 (↓)	0.4861	0.5525	0.5900	0.5019 (↓)
	s4	↓	↓	↓	↓	—	0.4523	0.3410	0.4014 (↓)	0.5525	0.5900	0.4777 (↓)
	s5	↓	↓	↓	↓	↓	0.4523	0.3410	0.4014	0.5525	0.4609 (↓)	0.4549 (↓)
*S*_4_		*F*_4_	*F*_3_	*F*_5_	*F*_23_	*F*_19_						
	base	—	—	—	—	—	0.6193	0.5775	0.5793	0.5699	0.5954	0.5867
	s1	↓	—	—	—	—	0.6193	0.3457 (↓)	0.5793	0.5699	0.5954	0.5641 (↓)
	s2	↓	↓	—	—	—	0.6193	0.3457	0.5793	0.5699	0.4862 (↓)	0.5414 (↓)
	s3	↓	↓	↓	—	—	0.4736 (↓)	0.3457	0.5793	0.5699	0.4862	0.5173 (↓)
	s4	↓	↓	↓	↓	—	0.3261 (↓)	0.3457	0.5793	0.5699	0.4862	0.4929 (↓)
	s5	↓	↓	↓	↓	↓	0.3261	0.3457	0.5003 (↓)	0.5699	0.4862	0.4708 (↓)

Note: the ↓ indicates a decrease.

**Table 10:** Robustness analysis under alternative modeling assumptions.

Robustness Test	Alternative Setting	*S*_1_	*S*_2_	*S*_3_	*S*_4_	Scenario Ranking	Stability Indicator
Baseline	VPN = 6; 5%; TN	0.5479	0.5631	0.5723	0.5867	*S*_4_ > *S*_3_ > *S*_2_ > *S*_1_	—
Coupling	VPN = 9	0.5512	0.566	0.5748	0.5894	*S*_4_ > *S*_3_ > *S*_2_ > *S*_1_	Top-3 overlap: 10/12
Coupling	VPN = 12	0.5438	0.5597	0.5688	0.5829	*S*_4_ > *S*_3_ > *S*_2_ > *S*_1_	Top-3 overlap: 9/12
Coupling	Exceedance = 2.5%	0.5496	0.5648	0.5737	0.5885	*S*_4_ > *S*_3_ > *S*_2_ > *S*_1_	Top-3 overlap: 11/12
Coupling	Exceedance = 10%	0.5451	0.5609	0.57	0.5842	*S*_4_ > *S*_3_ > *S*_2_ > *S*_1_	Top-3 overlap: 10/12
Root distribution	Beta	0.5416	0.5587	0.5689	0.5825	*S*_4_ > *S*_3_ > *S*_2_ > *S*_1_	Max. deviation < 1.2%
Expert parameters	*μ_i_* ± 10%, 100 runs	0.5483 *	0.5635 *	0.5727 *	0.5870 *	96/100 unchanged	* Mean across runs
Weights	*ω,η* ± 10%, 100 runs	0.5480 *	0.5632 *	0.5724 *	0.5868 *	100/100 unchanged	*ρ* = 0.88–0.92
Weights	*ω,η* Equal weighting	0.5526	0.5682	0.5744	0.5883	*S*_4_ > *S*_3_ > *S*_2_ > *S*_1_	Max. deviation = 0.90%

Note: * indicates statistical significance.

## Data Availability

The data presented in this study will be available on request. The data are not publicly available due to the data privacy policy implemented by the organization that funded the research.
